# Graph-Enhanced Retrieval-Augmented Generation for Postoperative Patient Question Answering: A Comparative Evaluation of Knowledge Graph, Retrieval-Augmented Generation, and Hybrid GraphRAG Approaches

**DOI:** 10.3390/bioengineering13101097

**Published:** 2026-09-22

**Authors:** Srinivasagam Prabha, Bernardo G. Collaco, Carina Rosa Malena, Syed Ali Haider, Nadia Wood, Antonio Jorge Forte

**Affiliations:** 1 Division of Plastic Surgery, Mayo Clinic, 4500 San Pablo Rd S, Jacksonville, FL 32224, USA; srinivasagam.prabha@mayo.edu (S.P.); collaco.bernardo@mayo.edu (B.G.C.); malenamonari.carina@mayo.edu (C.R.M.); haider.syedali@mayo.edu (S.A.H.); 2 Department of Radiology AI IT, Mayo Clinic, Rochester, MN 55905, USA; wood.nadia@mayo.edu; 3 Department of Artificial Intelligence and Informatics, Mayo Clinic, Jacksonville, FL 32224, USA; 4 Center for Digital Health, Mayo Clinic, Rochester, MN 55905, USA

**Keywords:** knowledge graph, retrieval-augmented generation, GraphRAG, postoperative care, patient communication, large language models, clinical AI, faithfulness, hallucination, surgical informatics

## Abstract

Background: Reliable access to postoperative guidance between clinic visits remains inconsistent, and the usefulness of large language models depends on how clinical knowledge is structured and retrieved. Knowledge graph-only (KG-only), vector retrieval-augmented generation (RAG-only), and hybrid graph-retrieval (GraphRAG) architectures have not been directly compared under controlled conditions for postoperative care. Objective: It was to compare these three pipelines on a curated postoperative question-answering benchmark using automated grounding metrics and blinded clinician review. Methods: A benchmark of 250 expert-authored questions (200 in-scope, 20 emergency-intent, 30 out-of-scope) was evaluated on an identical knowledge corpus using Gemini 2.5 Flash for generation and Gemini Embedding-001 for retrieval. Three blinded clinicians rated all 750 outputs; the primary outcome was majority-consensus accuracy across all 250 questions. GraphRAG is a multi-component pipeline that also includes query expansion, a second retrieval stage, evidence fusion, and a monotonic RAG fallback, and is therefore evaluated as a complete hybrid system rather than as graph retrieval in isolation. Results: Overall accuracy was 96.8% (95% CI: 93.8–98.4) for GraphRAG, 94.8% (95% CI: 91.3–96.9) for RAG-only, and 56.0% (95% CI: 49.8–62.0) for KG-only. Both retrieval-augmented systems outperformed KG-only (adjusted *p* < 0.001). The GraphRAG versus RAG-only difference was 2.0 percentage points, arose from five discordant question pairs and did not reach the Bonferroni-adjusted threshold (exact McNemar *p* = 0.062; adjusted *p* = 0.188). The monotonic RAG fallback determined 44.8% of GraphRAG outputs (112 of 250 queries). GraphRAG showed higher context recall (0.851 versus 0.796; adjusted *p* < 0.001), whereas RAG-only showed higher context precision. Estimated hallucination risk, derived from an LLM-as-a-judge procedure using Gemini 2.5 Flash rather than independent clinical adjudication, was 0.108, 0.132, and 0.436, respectively; the GraphRAG versus RAG-only difference was not significant (adjusted *p* = 0.771). Inter-rater agreement was high (Fleiss’ kappa = 0.854). Conclusions: Retrieval augmentation, rather than the choice of retrieval architecture, accounted for most of the difference observed. The incremental advantage of GraphRAG over a well-implemented RAG-only system was small, was not statistically significant after correction and reflects the complete hybrid system rather than graph augmentation alone. Emergency-intent accuracy was 75.0% for both retrieval-augmented systems; the architecture is therefore suited to supervised clinical workflows with clinician review, not autonomous patient-facing triage. Prospective validation on authentic patient messages and formal component ablations are required.

## 1. Introduction

Surgery marks the beginning of a recovery trajectory that unfolds largely at home, where patients must interpret and apply a dense body of clinical guidance received at discharge, often within hours of receiving an anesthetic and frequently while managing significant pain or fatigue. The instructions governing wound care, activity restrictions, medication schedules, and the warning signs that should prompt urgent clinical attention are critical, yet notoriously difficult for patients to retain and act upon reliably [1,2]. When questions arise in the days following discharge, as they invariably do, most patients lack access to a timely, knowledgeable source of advice outside of scheduled clinic hours. The resulting delays—whether from warranted clinical concern going unrecognized or from avoidable emergency department visits driven by unanswered anxiety—represent a persistent and addressable gap in the quality of postoperative care [3,4].

The growth of patient portal platforms has shifted some of this communication into asynchronous messaging channels, but the staffing burden of answering high volumes of postoperative messages in a clinically useful timeframe is considerable and often unmet [5,6]. Large language models (LLMs) have attracted significant interest as a potential mechanism for narrowing this gap [7,8,9]. More broadly, artificial intelligence has been framed as a way to augment clinical judgment and improve the delivery of medical care rather than replace clinicians [10]. The primary concern, however, is not what an LLM knows, but rather what it generates when its knowledge is incomplete: Hallucination—the production of confident but factually unsupported responses—remains a substantive and well-documented limitation of these models [11,12]. In a general-purpose setting, a hallucinated answer may merely be embarrassing; in postoperative care, where a patient may act on incorrect guidance regarding medication dosages, wound management, or symptom escalation, a plausible but inaccurate response can cause direct clinical harm [13,14].

Retrieval-augmented generation (RAG) was developed to address this limitation by anchoring LLM responses to retrieved documentary evidence rather than relying solely on parametric knowledge [15,16]. For clinical applications where an authoritative knowledge base can be constructed, RAG offers a practical pathway to grounded, source-attributed responses [17,18]. In practice, standard dense vector retrieval ranks chunks of text by embedding similarity—an approach that captures semantic proximity well but does not explicitly represent the typed, relational structure of clinical knowledge [19]. Knowledge graphs (KGs) can encode exactly this kind of relational information: typed entity nodes connected by labelled edges, forming a structured network of clinical relationships with explicit provenance [20,21]. However, their acknowledged limitation is an extreme sensitivity to query terminology. A patient asking “can I have a glass of wine?” or “is it okay to drive?” may not phrase their query in language that maps easily onto the canonical entity vocabulary of even a well-constructed graph [22,23].

GraphRAG combines both strategies, structured KG triple retrieval and dense vector chunk retrieval into a single hybrid pipeline [24,25]. We use the term GraphRAG narrowly in this manuscript to refer to a hybrid pipeline in which a structured knowledge graph and a dense vector index are queried in parallel, and their retrieved evidence is merged before generation. Related but architecturally distinct approaches—such as knowledge-graph-augmented retrieval (where the KG is used only to expand or filter dense retrieval rather than acting as a parallel evidence source), ontology-guided retrieval (where queries are mapped to a controlled vocabulary before retrieval), and community-detection-based GraphRAG [24] (where hierarchical graph summaries replace document chunks)—share conceptual ground but differ materially in their retrieval mechanisms and evaluation profiles. Consequently, we make no claims about those alternative approaches beyond the specific hybrid pipeline evaluated here.

Empirical support for this hybrid premise has emerged from several biomedical settings, although the evidence remains preliminary and existing studies differ widely in clinical tasks, knowledge bases, and evaluation designs. Song and colleagues reported that graph-augmented retrieval improved diagnostic coverage in rare genetic disease question answering by more than 25 percentage points over an unaugmented baseline, although they noted that vector retrieval generally outperformed structured graph queries when used in isolation [26]. Liu and colleagues reported near-perfect sensitivity and specificity for emergency classification in patient portal messages when using a knowledge-graph-based RAG pipeline with hierarchical community summarization [27]. Structured reflective reasoning over medical knowledge graphs has also been shown to improve answer faithfulness in specialty-specific decision support [28]. Related work on knowledge-infused prompting has similarly shown that incorporating structured clinical knowledge into LLM generation can improve the quality and clinical grounding of generated text [29]. While these studies provide directional support for the value of graph augmentation, they have not been compared against each other or against their constituent components under matched clinical conditions. Furthermore, the sheer heterogeneity of their tasks and metrics complicates any strong generalization about the fundamental superiority of graph-augmented retrieval over well-implemented dense retrieval. The marginal contribution of architectural choice relative to retrieval design, prompt engineering, and knowledge-base quality remains an open empirical question.

The present study directly addresses this gap. We implemented KG-only, RAG-only, and GraphRAG pipelines on an identical postoperative knowledge corpus using the same backbone language model and the same embedding model, fixing all retrieval and generation parameters before evaluation began. We then evaluated all three systems against a 250-question expert-authored benchmark spanning 28 clinical and out-of-scope categories. All 750 outputs were independently rated by three blinded clinicians, supplemented by automated retrieval-grounding metrics and a binary-normalized hallucination panel.

To characterize performance across the native-hybrid and RAG-fallback strata within the GraphRAG pipeline, we provide an analytical decomposition of observed GraphRAG output performance in Section 3.11. This decomposition uses the fact that, by design, the monotonic fallback substitutes the corresponding RAG-only response verbatim. The resulting native-hybrid estimate is therefore conditional on the stated decomposition assumption and should not be interpreted as an experimental isolation of the knowledge-graph component or as a substitute for formal ablation analysis.

The novelty of the present work is fivefold. First, it shifts the clinical task from diagnosis or emergency classification to patient-facing postoperative guidance, where a safe answer requires the complex integration of symptoms, procedures, postoperative timing, self-care instructions, and escalation criteria [30,31]. Second, it uses a controlled, matched three-arm design, KG-only versus RAG-only versus GraphRAG, with a shared knowledge base and an identical generation model, to compare structured graph retrieval, dense text retrieval, and their fusion under matched conditions. We emphasize that the GraphRAG arm is a multi-component pipeline that additionally incorporates query expansion, a second retrieval stage, evidence fusion, and a monotonic RAG fallback. The design therefore compares complete architectures and does not isolate the marginal contribution of the graph component itself; formal component ablations remain necessary and are identified as a principal limitation of this work. Third, it embeds explicit safety routing, monotonic RAG fallback logic, and reflective repair into the retrieval-generation pipeline, enabling the system to abstain, refuse out-of-scope queries and escalate emergency-sensitive presentations [32,33]; emergency handling was nevertheless imperfect in this benchmark and is reported as a safety limitation rather than a validated capability. Fourth, it introduces an analytical decomposition framework for hybrid retrieval that partitions observed aggregate performance into native-hybrid and RAG-fallback strata, providing a descriptive characterization of system behavior while explicitly acknowledging that the decomposition does not isolate component-level causal effects. Fifth, it evaluates patient-facing readability alongside clinical accuracy and safety using Flesch Reading Ease and Flesch−Kincaid Grade Level, adding a patient-centered communication dimension relevant to deployment in low-health-literacy populations. A visual overview of the study design is shown in Figure 1, and the comparative methodology of the three pipelines is summarized in Figure 2.

## 2. Materials and Methods

### 2.1. Study Design

This was a comparative benchmark evaluation study designed to characterize the performance of three retrieval-generation architectures on a standardized postoperative clinical question answering task. The three systems, namely KG-only, RAG-only, and GraphRAG, were fully implemented and their parameters fixed before evaluation commenced. All three pipelines used the same language model for answer generation and the same evaluation workflow. The same embedding model was used wherever dense retrieval was required, and all pipelines followed an identical response policy for emergency escalation and out-of-scope refusal.

### 2.2. Dataset and Evaluation Set Composition

The evaluation set comprised 250 questions constructed by the clinical study team to represent the informational landscape of the postoperative period. The set was designed with a fixed, pre-specified category structure: 200 in-scope routine postoperative queries distributed evenly across 20 predefined care categories at 10 questions per category (pain management, wound and drain care, diet, medication interactions, activity and lifting, nausea and vomiting, showering and bathing, sleep disturbance, support garments, sexual activity, sutures, scar, return to work, travel and driving, emotional wellbeing and body image, need for home assistance, follow-up, recovery timeline, alarm signs, and additional treatments); 20 emergency-intent questions carrying escalation-sensitive signals; and 30 out-of-scope queries spanning seven non-clinical domains (entertainment, finance, general knowledge, lifestyle, technology, travel, and weather). Ground-truth reference answers for all in-scope questions were compiled from the knowledge corpus content by a clinical team member and served as the reference standard for automated metric computation; where source documents disagreed, the study team resolved the discrepancy by consensus in favor of the most recent evidence-backed guidance, as described in Section 2.9. The operational criteria used to judge routine, emergency-intent, and out-of-scope responses are specified in Section 2.9, and the complete category-level composition of the evaluation set is summarized in Table 1. The questions were authored specifically for this study rather than drawn from patient portal traffic; the detailed authoring workflow, including any grading of question difficulty, independent re-review of individual items, and formal de-duplication screening, was not prospectively documented, and this is acknowledged as a limitation in Section 4.10.

### 2.3. Knowledge Base and Document Preparation

All three systems operated over a shared postoperative care knowledge corpus stored as a structured plain-text document. The corpus contained discharge instruction content, wound care protocols, activity and lifting guidance, medication management information, drain care, follow-up scheduling, and escalation criteria for postoperative complications. The document was segmented into overlapping text chunks of maximum 1400 characters with a one-sentence overlap between consecutive segments. Embedding vectors were generated using the Gemini Embedding-001 model and stored as a matrix indexed by chunk identifier. The full pipeline overview is shown in Figure 1.

### 2.4. KG-Only Pipeline

The KG-only pipeline derived answers exclusively from a lightweight domain knowledge graph constructed directly from the text corpus, without dependence on external ontologies or pre-existing biomedical databases. Graph construction identified eight predefined node categories: Symptom, RedFlag, Complication, Procedure, Medication, Self Care Action, Outcome, and TimeWindow. Five typed relation categories were used: Expected_After, Requires_Escalation_To, Managed_With, Contraindicated_With, and Related_To. Forward and reverse adjacency lists were maintained for all edges, enabling bidirectional traversal from any query-seeded node. At inference time, queries were canonicalized, classified and used to seed multi-hop graph traversal. Two-hop traversal was selected because postoperative reasoning often requires linking a symptom to a complication and then to an escalation action; one-hop retrieval may miss clinically important paths, whereas deeper traversal beyond two hops risks introducing weakly related evidence. The complete KG-only pipeline is formalized in Algorithm 1 [19,20,23].

We note that the KG used here is a corpus-derived rule-based construction, not an ontology-aligned graph. We deliberately did not align the KG to SNOMED CT or UMLS for two reasons: first, to keep the three-arm comparison genuinely controlled (the same source corpus, same backbone model, same infrastructure); second, to mirror pragmatic clinical deployment constraints, as the vast majority of local institutional postoperative care databases are not pre-annotated to a standard biomedical ontology. The performance reported here for KG-only therefore represents the floor of what a domain-specific, corpus-derived KG retrieval system can achieve; ontology alignment would likely raise this floor and is explicitly discussed as a future direction in Section 4.11 [34].
**Algorithm 1.** KG-only pipeline (end-to-end question answering)Input:       User query q. Postoperative care knowledge graph G.                    Generation model M (Gemini 2.5 Flash, temperature = 0.1). Output:    Final answer bundle: answer text, confidence, abstention flag, emergency es                   calation flag.Step 1.      Query planning: classify question type; extract canonical domain terms; detect                   temporal context; evaluate emergency or out-of-scope patterns. Step 2.      Safety routing: emergency → escalation; out-of-scope → decline. Step 3.      Seed node identification: match canonical query terms to G. Step 4.      One-hop traversal: collect connected triplets; score by base                  one-hop weight + clinical-salience + query overlap. Step 5.      Two-hop traversal: discounted base weight; exclude loops. Step 6.      Fact ranking: merge, dedupe, top 16. Step 7.      Context assembly: source-chunk recovery via provenance. Step 8.      Answer generation: evidence-only prompt; structured JSON. Step 9.      Reflective repair: cautious retry if abstained with evidence;                  reanswer once if confidence below threshold. Step 10.    Return final answer bundle.

### 2.5. RAG-Only Pipeline

The RAG-only pipeline retrieved generation context exclusively through dense vector similarity. At inference time, the incoming query was embedded using Gemini Embedding-001 and cosine similarity computed between the query vector and all stored chunk embedding vectors. The top eight chunks by similarity score formed the generation context. No graph-derived evidence was incorporated. The generation prompt, response format, false-abstention reduction mechanism, and emergency and out-of-scope routing were identical to those applied in the KG-only pipeline. The complete RAG-only pipeline is formalized in Algorithm 2 [14,16].
**Algorithm 2.** RAG-only pipeline (end-to-end question answering)Input:       User query q. Embedding matrix E (Gemini Embedding-001).                  Generation model M. Output:      Final answer bundle.Step 1.      Query planning (as in Algorithm 1). Step 2.      Safety routing (as in Algorithm 1). Step 3.      Query embedding → v_q. Step 4.      Cosine similarity over E. Step 5.      Top-k = 8 chunks; optional lexical rescoring. Step 6.      Context assembly; no graph evidence. Step 7.      Answer generation (as in Algorithm 1). Step 8.      Reflective repair (as in Algorithm 1). Step 9.      Return final answer bundle.

Implementation environment and storage design were performed. All three systems were implemented in the same computational environment using Google Cloud Vertex AI Workbench (Google LLC, Mountain View, CA, USA; Workbench notebook image workbench-notebooks:m139) with Python 3.10.19. The computational workload was executed in the us-central1 Google Cloud region. The implementation used the Vertex AI SDK for Python, together with NumPy, pandas, and tqdm. To preserve a controlled comparison across retrieval architectures, all three systems used the same source corpus, generation model, and evaluation workflow, with the same embedding model wherever dense retrieval was required. The KG component was implemented as a lightweight corpus-derived graph using JSON-based artifacts and in-memory adjacency maps, while the RAG component used a local embedding matrix and chunk metadata rather than an external vector database. This design minimized infrastructure-related confounding and allowed the evaluation to focus on retrieval strategy rather than differences in database backends, indexing systems, or deployment infrastructure.

### 2.6. GraphRAG (Hybrid KG + RAG) Pipeline

The GraphRAG pipeline combined graph-derived relational evidence with dense semantic retrieval through parallel execution, graph-guided retrieval expansion, adaptive graph suppression, time-aware fact selection, evidence fusion, and a monotonic RAG fallback. The complete procedure is formalized in Algorithm 3. The comparative methodology of all three architectures is illustrated in Figure 2.

Retrieval was initiated in parallel for each query, producing an initial pool of graph facts with linked chunks and a separate pool of semantically retrieved text chunks. Subject and object entity terms from the retrieved graph facts were extracted, cleaned of stopwords and domain-generic placeholder terms, and used to construct an expanded retrieval query oriented toward entity-specific content. When graph facts were too sparse—fewer than two temporally relevant facts and fewer than three total facts—and no KG-linked chunks were available, graph evidence was suppressed. For timing- or instruction-oriented queries, a time-aware fact selection step preferentially included graph facts containing temporal markers.

A set of hard rules implemented the monotonic RAG fallback and regulated final answer selection. For critical clinical domains, including postoperative timelines, direct surgical instructions, symptom management, and medication guidelines, if the hybrid pipeline abstained from generating a response despite a valid RAG-only answer being available, the RAG-only output was substituted verbatim. This fallback was designed to reduce the risk that the hybrid system would perform worse than the dense retrieval baseline when graph-derived evidence was sparse or unhelpful. Methodologically, this means that a proportion of the final GraphRAG outputs are identical to RAG-only outputs; the frequency of fallback activation and its impact on performance are detailed in Section 3.8 and Section 3.11 [25].
**Algorithm 3.** GraphRAG hybrid pipeline (end-to-end question answering)Input:      User query q. Knowledge graph G. Embedding matrix E.                  Corpus chunk store. Generation model M. Output:    Final answer bundle.Step 1.      Query planning (as in Algorithm 1). Step 2.      Safety routing (as in Algorithm 1). Step 3.      Parallel retrieval: KG traversal + dense retrieval. Step 4.      Graph-guided expansion: entity-anchored second retrieval. Step 5.      Adaptive graph suppression (utility threshold). Step 6.      Time-aware fact selection (temporally-marked priority). Step 7.      Context window assembly: merge, dedupe, rank, top 10. Step 8.      Answer generation (as in Algorithm 1). Step 9.      Reflective repair (as in Algorithm 1). Step 10.    Monotonic RAG fallback: substitute RAG-only answer if                  hybrid abstains and RAG-only scores higher. Step 11.    Return final answer bundle.

#### Safety and Scope-Routing Policy

All three pipelines used the same prompt-level safety and scope policy. For the 200 routine in-scope postoperative questions, the systems were instructed to answer using only retrieved evidence from the postoperative knowledge corpus and to avoid unsupported medical advice. For the 20 emergency-intent questions, the systems were instructed not to provide routine postoperative guidance and instead to direct the user to seek urgent clinical help or emergency services, such as calling emergency services or contacting the surgical team immediately, depending on the severity of the presentation. For the 30 out-of-scope questions, the systems were instructed to politely decline the request and explain that the assistant was limited to postoperative recovery guidance. This shared policy was applied across KG-only, RAG-only, and GraphRAG, so that differences in performance reflected retrieval architecture and safety-boundary handling rather than different prompt instructions.

### 2.7. Hyperparameter Configuration

Hyperparameters were selected using a 50-question calibration subset drawn from the 250-question benchmark. After calibration, all parameters were frozen and the three systems were evaluated across the complete 250-question set. Deterministic decoding was used (temperature: 0.1, top-p: 1.0, candidate count: 1, max output: 2048 tokens). The final configuration was as follows: chunk size: 1400 characters; chunk overlap: one sentence; RAG top-k = 8; KG max facts = 16; KG seed nodes = 4; KG one-hop facts = 8; KG two-hop facts = 6; KG-linked snippets = 8; one-hop base score: 1.00; two-hop base score: 0.65; reflection repair rounds = 1. A sensitivity analysis varying rag_top_k ∈ {5, 8, 10, 12}, KG graph depth ∈ {1, 2, 3}, and decoding temperature ∈ {0.0, 0.1, 0.2} was performed on the calibration subset; results were stable across these perturbations and the final values reported above were selected for the primary benchmark.

### 2.8. Automated Evaluation Metrics

Automated evaluation was conducted using the RAGAS (Retrieval-Augmented Generation Assessment) framework. We calculated three validation metrics: context precision, context recall, and answer semantic similarity. Each metric is bounded between 0 and 1, with higher values indicating superior performance [35].

A methodological distinction between precision metrics was made. To prevent conceptual confusion, we explicitly distinguished between classification precision and context precision. Classification Precision is an answer-level metric derived from the clinician-rated confusion matrix. It quantifies the proportion of system-generated answers that were judged clinically accurate by blinded reviewers, conditioned on the system having produced a response. Context Precision is a RAGAS retrieval-level metric. It quantifies the proportion of retrieved document segments that were relevant to both the user query and the ground-truth reference answer [35].

These two metrics evaluate distinct components of the generation pipeline and may yield different architectural rankings. For example, the KG-only pipeline often achieves high classification precision because it generates answers only when highly confident graph facts are matched, despite a high rate of clinical abstentions (low recall). Conversely, the RAG-only pipeline optimizes semantic retrieval to maximize context precision but may have weaker boundary safety on out-of-scope queries (reducing classification precision). The hybrid GraphRAG pipeline merges both modalities; this broadens the retrieved evidence window—which can marginally reduce context precision—while significantly improving context recall and providing the generator with a richer context package. We report both metrics to isolate retrieval-level evidence quality from final generative correctness.

#### 2.8.1. Concordance Between Automated and Clinician Evaluations

We evaluated the convergent validity of the automated safety panel against the blinded clinician classifications at the system level. Because the clinical reviewers utilized binary accuracy classifications alongside Likert-scaled completeness and Sensibility–Specificity–Interestingness (SSI) relevance, while the LLM-as-judge operated on a distinct 1–5 scoring rubric, direct case-level Spearman rank correlations could not be calculated in a scale-matched manner.

Instead, we assessed concordance via the aggregate performance rankings of the three pipeline architectures across both evaluation streams. The retrieval-augmented systems consistently outperformed KG-only across the automated safety panel. RAG-only and GraphRAG showed broadly similar performance, with GraphRAG achieving the most favorable values on most, but not all, automated dimensions, two of the three RAGAS metrics, and all three clinician-rated dimensions (accuracy, completeness, and relevance). This overall alignment provides convergent support for the consistency of the automated evaluation framework.

#### 2.8.2. Safety and Hallucination Evaluation (LLM-as-a-Judge)

To systematically evaluate safety and hallucination profiles, we implemented a structured LLM-as-a-judge panel. All 750 generated outputs were evaluated by Gemini 2.5 Flash (operating in deterministic mode: temperature = 0.1, top-p = 1, max tokens = 2048) across six key dimensions: factual correctness, faithfulness, completeness, safety, hallucination risk, and an overall hallucination-free score.

To minimize scoring variance and control for potential stylistic or structural biases, all three pipeline outputs (KG-only, RAG-only, and GraphRAG) for any given query were evaluated simultaneously in a single API call, anchored against the same ground-truth clinical reference.

The evaluation prompt explicitly instructed the judge to the following: rely exclusively on the provided expert reference answer, accept clinically equivalent paraphrasing and medical terminology, evaluate the omission of reference information strictly under the completeness metric and restrict hallucination classification to instances where the system introduced active fabrications, contradictions, clinically misleading details, or unsafe medical advice unsupported by the reference. Scores were recorded on a 1–5 Likert scale, normalized to a 0–1 scale and subsequently binarized using a threshold of ≥0.5 to yield the binary safety panel metrics reported in Section 3.7 [36].

#### 2.8.3. Analytical Decomposition of GraphRAG Component Contributions

Because the monotonic RAG fallback within the GraphRAG pipeline substituted the RAG-only response verbatim when triggered, the final GraphRAG output stream can be partitioned mathematically into two distinct strata: the fraction of queries where the fallback was triggered (rendering the output identical to the RAG-only baseline) and the complementary fraction of queries generated natively by the hybrid graph-retrieval pipeline.

This architectural configuration allowed us to analytically estimate performance within the native-hybrid stratum from the observed aggregate statistics under the stated decomposition assumption, without requiring a separate physical ablation arm during the blinded clinician evaluation. The mathematical decomposition is detailed in Section 3.11.

### 2.9. Human Expert Evaluation

Clinical accuracy was evaluated using a binary score of correct (1) or incorrect (0). The expert-authored reference answers were extracted directly from the shared postoperative knowledge corpus used to anchor the retrieval architectures. If discrepancies arose between source documents, the study team resolved them by consensus, giving priority to the most recent, evidence-backed clinical guidelines. For each system output, the final binary label was determined by majority clinician agreement; a correct classification required at least two of the three blinded reviewers to rate the response as correct. To measure consistency across this evaluation panel, inter-rater reliability was calculated using Fleiss’ kappa and pairwise Cohen’s kappa with bootstrap 95% confidence intervals derived over 2000 iterations, with scores interpreted using the Landis and Koch thresholds [37].

Accuracy was assessed separately for standard clinical queries and safety-sensitive boundary cases. The evaluation benchmark comprised 250 queries: 200 in-scope postoperative questions, 20 emergency escalation scenarios, and 30 deliberately out-of-scope prompts unrelated to surgical recovery. Clinical Medical Accuracy was evaluated across the 220 clinical questions (comprising the 200 in-scope queries and the 20 emergency scenarios), measuring whether the system provided a clinically sound, correct answer. Safety/Refusal Accuracy was evaluated across the 30 out-of-scope prompts, measuring whether the system successfully refused to answer or recognized that the query fell outside the intended postoperative support domain.

Evaluation of emergency and out-of-scope prompts: Emergency-intent prompts were considered correctly handled when the system recognized the escalation-sensitive nature of the query and advised urgent clinical or emergency care rather than providing routine self-management advice. Out-of-scope prompts were considered correctly handled when the system refused or redirected the query by stating that it could only answer postoperative recovery-related questions. Routine in-scope prompts were considered correct when the response provided clinically appropriate postoperative guidance grounded in the knowledge corpus.

To capture comprehensive system performance across the entire 250-query benchmark, a global confusion-matrix framework was established (Figure 3). Under this framework, correct answers to clinical questions (both routine and emergency) were classified as true positives (TPs), and any incorrect or failed answers were classified as false negatives (FNs). For out-of-scope queries, appropriate refusals were classified as true negatives (TNs), while inappropriate or attempted answers were marked as false positives (FPs). These classifications formed the basis for calculating overall accuracy, precision, recall, and F1 scores.

For queries involving patient-specific logistics that require individualized context—such as specific appointment times, medication adjustments, or surgeon-dependent recovery plans—responses were marked correct if the system clearly stated its lack of access to private data and advised the user to consult their surgical team. This prevented pipelines from being penalized for safely identifying information gaps. Robustness of these clinician evaluations was verified via sensitivity analysis by recalculating accuracy scores under a strict, unanimous consensus rule requiring agreement among all three reviewers.

Completeness of each response was graded on a 5-point Likert scale: A score of 1 (strongly incomplete) indicated that the response missed almost all key clinical elements needed to address the question safely. A score of 2 (major omissions) reflected substantial omissions of key clinical components. A score of 3 (partial coverage) indicated that some relevant facts were present, but important details were missing. A score of 4 (near-complete) indicated the denoted near-complete coverage with only minor clinical omissions. A score of 5 (strongly complete) represented a fully comprehensive response aligned with the reference standard. The final completeness rating for each output was calculated as the mean score across the three reviewers.

Relevance was quantified using the Sensibility–Specificity–Interestingness (SSI) Index on a 0–3 scale. One point was awarded for each met criterion: The response had to be sensible, specific to the clinical prompt and contextually useful. The final relevance score represented the sum of these three binary ratings. This index isolated conversational flow, coherence, and specificity, while actual clinical safety and correctness were captured independently by the binary medical accuracy metric.

### 2.10. Statistical Analysis

All statistical analyses were executed in Python 3.10.19 using the SciPy, statsmodels, and scikit-learn libraries. Individual tests were two-sided, using a baseline significance threshold of α = 0.05 established prior to multiple-comparison corrections.

Overall binary accuracy was evaluated using Cochran’s Q test [38], while continuous outcomes were compared using Friedman’s test [39]. For subsequent pairwise analyses, binary outcomes were compared using the exact two-sided McNemar test; the mid-P variant was additionally reported as a sensitivity analysis. Wilcoxon signed-rank tests were applied to continuous scores [40,41]. Bonferroni corrections adjusted all pairwise *p*-values, and proportions are reported with 95% Wilson confidence intervals [42]. To assess primary safety improvements, bootstrap 95% confidence intervals were generated using the percentile method over 10,000 iterations, comparing GraphRAG’s hallucination risk directly against the KG-only and RAG-only baselines. A post-hoc power consideration was performed for the primary pairwise accuracy comparison between RAG-only and GraphRAG. Given n = 250 questions and an observed overall accuracy difference of 2.0 percentage points, the pairwise comparison did not reach the Bonferroni-adjusted significance threshold. Because the absolute difference was modest and the odds-ratio confidence interval was wide, the result should be interpreted as an imprecise, underpowered signal in the direction of GraphRAG rather than definitive evidence of broad superiority.

To assist with clinical interpretation, corresponding effect sizes accompany every reported value. Kendall’s W served as the effect size for the continuous Friedman tests. For categorical data analyzed via Cochran’s Q, the equivalent effect size was calculated using the formula: W = Q/[n (k − 1)], where Q is the Cochran’s Q test statistic, n is the number of query items, and k is the number of groups. Pairwise Wilcoxon signed-rank effect sizes were reported as r = |Z|/√N, where N is the number of paired observations. Pairwise McNemar comparisons are summarized using odds ratios alongside their 95% confidence intervals. Inter-rater agreement (both Fleiss’ and Cohen’s kappa) was mapped to the standard −1.0 to +1.0 scale, with bootstrap 95% confidence intervals derived over 2000 iterations and interpreted using the Landis and Koch framework [37]. Finally, the Haldane–Anscombe correction, adding 0.5 to each discordant cell, was applied to odds-ratio estimates when either discordant cell was zero.

### 2.11. Ethical Considerations

No patient-level medical records, personal identifiers, or clinical interventions were utilized in this study. Our evaluation set consisted entirely of custom questions and reference answers written by our clinical team based on public, general postoperative recovery guidelines. No electronic health records, clinician communications, or protected health information were accessed at any stage. Clinical reviewers participated as members of the research team, scoring system outputs directly against the prepared reference standards. Because the study involved no actual patients, patient consent was not required, no financial compensation was provided, and no patient clinical outcomes were tracked.

To support safety, all systems included prompt-level instructions for emergency escalation and out-of-scope refusal. Emergency-intent queries were expected to trigger urgent-care guidance rather than routine postoperative advice, and out-of-scope queries were expected to receive a polite refusal or redirection to the postoperative domain. These safeguards were evaluated explicitly rather than assumed to be uniformly successful. Because the study utilized no patient-identifiable data and involved no human subjects, formal Institutional Review Board review was not required under applicable institutional guidelines.

## 3. Results

### 3.1. Dataset Composition and Evaluation Design

The evaluation benchmark comprised 250 questions distributed across 28 categories: 20 routine postoperative categories (n = 200 questions, 80.0%), 7 out-of-scope categories (n = 30 questions, 12.0%), and 1 emergency-intent category (n = 20 questions, 8.0%); the full breakdown is shown in Table 1. All 750 model outputs (250 questions × 3 systems) were evaluated by three independent blinded clinical reviewers under matched conditions, with the evaluation order randomized.

### 3.2. Primary Accuracy: Overall Human-Rated Performance

Overall accuracy differed substantially across the three systems. Under the primary majority-consensus criterion applied to all 250 benchmark questions, KG-only achieved 56.0% (95% CI: 49.8–62.0%), RAG-only achieved 94.8% (95% CI: 91.3–96.9%), and GraphRAG achieved 96.8% (95% CI: 93.8–98.4%); the omnibus Cochran’s Q test was highly significant (Q = 188.06, df = 2, *p* < 0.001). All 250 questions reached majority reviewer consensus for every system, so no questions were excluded from the primary analysis. Disaggregated by question type, clinical medical accuracy across the 200 in-scope postoperative questions and 20 emergency-intent questions was 50.9% (95% CI: 44.3–57.4%) for KG-only, 96.4% (95% CI: 93.0–98.1%) for RAG-only, and 97.3% (95% CI: 94.2–98.7%) for GraphRAG. Safety/refusal accuracy across the 30 out-of-scope questions was 93.3% (95% CI: 78.7–98.2%) for KG-only, 83.3% (95% CI: 66.4–92.7%) for RAG-only, and 93.3% (95% CI: 78.7–98.2%) for GraphRAG. Accuracy on the 20 emergency-intent questions alone was 65.0% (95% CI: 43.3–81.9%) for KG-only and 75.0% (95% CI: 53.1–88.8%) for both RAG-only and GraphRAG. The subgroup counts reconcile exactly with the overall totals (112 + 28 = 140 for KG-only, 212 + 25 = 237 for RAG-only, and 214 + 28 = 242 for GraphRAG). The overall and subgroup accuracy results are summarized in Figure 4.

A note on the parametric LLM-only baseline was made. A separate parametric-LLM-only arm (Gemini 2.5 Flash with no retrieval) was not evaluated as part of this study. The decision to compare retrieval architectures against one another was made on the grounds that the clinical question for a deployment scenario is which retrieval pipeline is most appropriate, not whether retrieval contributes over parametric knowledge—the latter question has been answered repeatedly in the adjacent clinical AI literature [8,16,26,27]. This absence is acknowledged explicitly as a limitation in Section 4.10.

Subgroup interpretation was performed. The benchmark included 200 routine in-scope postoperative questions, 20 emergency-intent questions, and 30 out-of-scope questions. For the primary subgroup analysis, clinical medical accuracy was calculated across the 220 clinical/emergency questions, because both routine postoperative guidance and emergency escalation require clinically correct handling. Safety/refusal accuracy was calculated separately across the 30 out-of-scope questions, where the expected behavior was appropriate refusal or redirection rather than clinical answering. Overall accuracy was calculated across all 250 questions. This separation avoids conflating clinical answering performance with out-of-scope boundary handling and provides a clearer interpretation of system behavior across clinically distinct query types.

Means and 95% Wilson confidence intervals are reported for each method. Clinical medical accuracy was calculated across the 220 clinical/emergency questions, including 200 in-scope postoperative questions and 20 emergency-intent questions. Safety/refusal accuracy was calculated across the 30 out-of-scope questions. Overall accuracy was calculated across the full 250-question benchmark. Overall *p*-value was calculated using Cochran’s Q test.

Completeness and relevance ratings followed the same directional pattern as overall accuracy, but with even larger effect sizes. KG-only responses were rated as substantially incomplete by the blinded clinical panel, achieving a mean completeness score of 2.70 (SD: 1.44) on the 1–5 scale a score that reflects responses that covered only some key elements while omitting clinically important details (Figure 5; Table 2). By contrast, RAG-only (mean: 4.64, SD: 0.78) and GraphRAG (mean: 4.73, SD: 0.66) both clustered at the top of the scale with narrow distributions, indicating that retrieval-augmented outputs were consistently judged as covering all or nearly all relevant clinical content. The differences were highly significant (Wilcoxon signed-rank, both KG-only comparisons adjusted *p* < 0.001, r = 0.508; large effect), and RAG-only and GraphRAG did not differ meaningfully from each other (adjusted *p* = 0.087). Clinician-rated relevance, measured using the SSI index (0–3), showed the same pattern (Figure 6). KG-only responses were rated as frequently imprecise or off-target (mean: 1.84, SD: 0.85; median: 1.65), with scores spanning almost the entire scale, reflecting the system returning partially matched graph facts that did not fully address the patient’s specific query. RAG-only (mean: 2.88, SD: 0.37) and GraphRAG (mean: 2.91, SD: 0.33) both achieved near-ceiling relevance with very little variability, indicating that dense retrieval consistently anchored responses to the clinically appropriate content. Again, both retrieval-augmented systems significantly outperformed KG-only (both adjusted *p* < 0.001, r = 0.508), while the difference between RAG-only and GraphRAG was not statistically significant (adjusted *p* = 0.054). Taken together, the completeness and relevance results reinforce the primary accuracy findings: the failure of KG-only is not simply a matter of binary correctness but extends to the depth and specificity of clinical content provided, both of which are essential for a postoperative patient to act safely on the information received.

### 3.3. Pairwise Comparisons

Pairwise McNemar comparisons with Bonferroni correction (k = 3 comparisons per outcome) confirmed that both retrieval-augmented systems significantly outperformed KG-only on overall accuracy. The KG-only versus RAG-only odds ratio was 0.044 (95% CI: 0.017–0.114; adjusted *p* < 0.001), and the KG-only versus GraphRAG odds ratio was 0.014 (95% CI: 0.003–0.073; adjusted *p* < 0.001). The RAG-only versus GraphRAG comparison did not reach the Bonferroni-adjusted significance threshold. All 250 questions reached a majority reviewer consensus for both systems and were retained, so no pairs were excluded from this comparison. Of these, 237 questions were answered correctly by both systems and 8 were answered correctly by neither system. The discordant cells comprised 5 questions answered correctly by GraphRAG but not by RAG-only (n01 = 5) and none answered correctly by RAG-only but not by GraphRAG (n10 = 0). The paired absolute risk difference was therefore 2.0 percentage points in favor of GraphRAG (95% CI: 0.1 to 4.5 percentage points, Newcombe score method, unadjusted for multiplicity), with a Haldane−Anscombe-corrected odds ratio of 0.091 (95% CI: 0.005–1.644), an exact two-sided McNemar *p*-value of 0.062, and a Bonferroni-adjusted *p*-value of 0.188. The mid-P variant gave a raw *p*-value of 0.031 and an adjusted *p*-value of 0.094. The comparison therefore sits at the boundary of nominal significance before correction and does not reach significance after correction for three pairwise comparisons. Because the entire difference rests on five discordant question pairs, with one discordant cell empty, the interval estimate and the exact test are only marginally consistent; we therefore interpret this comparison as inconclusive rather than as demonstrating superiority of GraphRAG.

Power was considered. The observed overall accuracy difference between RAG-only and GraphRAG was 2.0 percentage points across the 250-question benchmark, arising from only five discordant question pairs. The benchmark was not powered to resolve a difference of this size: With five discordant pairs and none in the opposing cell, the exact McNemar test yields a raw *p*-value of 0.062, which cannot reach significance after Bonferroni correction for three pairwise comparisons per outcome. This comparison should therefore be interpreted as an underpowered and statistically inconclusive signal in the direction of GraphRAG, and not as evidence of either superiority or equivalence between the two retrieval-augmented systems. Determining whether a difference of this magnitude is real and clinically meaningful would require a substantially larger benchmark with a prespecified superiority or non-inferiority margin.

Full pairwise comparison statistics, including completeness, relevance, RAGAS, and hallucination panel metrics, are presented in Table S1.

OR indicates the odds ratio; CI indicates the confidence interval; FRE indicates the Flesch Reading Ease (higher = more readable); FKGL indicates the Flesch−Kincaid Grade Level (lower = more readable). An exact two-sided McNemar test was performed for binary accuracy comparisons; a mid-P McNemar test was reported as a sensitivity analysis; a Wilcoxon signed-rank test was performed for all continuous outcomes. All *p*-values were adjusted using Bonferroni correction (k = 3 pairwise comparisons per variable). Where either discordant cell (n10 or n01) was zero, a Haldane−Anscombe continuity correction (0.5 added to each discordant cell) was applied before computing ORs and 95% CIs. Effect size r was calculated as |Z|/sqrt(N) for Wilcoxon; ORs (95% CIs) were reported for McNemar. Effect size r was interpreted as follows: <0.1 negligible, 0.1–0.3 small, 0.3–0.5 moderate, ≥0.5 large. Clinical medical accuracy was assessed on 220 questions (questions 1–200 + emergency 231–250). Safety/refusal accuracy was assessed on 30 out-of-scope questions (out-of-scope questions: 201–230). RAGAS Answer correctness and faithfulness were excluded due to RAGAS API computation failures. *** *p* < 0.001; ** *p* < 0.01; * *p* < 0.05; ns means not significant (adjusted *p* > 0.05). Color bands: orange indicates the accuracy; green indicates the completeness; blue indicates the relevance; purple indicates RAGAS; pink indicates hallucination; teal indicates the readability.

### 3.4. Classification Metrics from the Confusion Matrix

Disaggregation of the TP/TN/FP/FN counts revealed the specific failure mode of each system (Table 3). KG-only produced 108 false negatives, representing 98.2% of its 110 incorrect classifications. In contrast, RAG-only and GraphRAG produced far fewer false negatives, with 8 and 6, respectively. False-positive responses to out-of-scope queries were uncommon across all systems but were higher for RAG-only (5) than for KG-only or GraphRAG (2 each). Using the combined clinical/emergency class as the positive class, classification precision was 0.982 for KG-only, 0.977 for RAG-only, and 0.991 for GraphRAG. Classification recall was 0.509, 0.964, and 0.973, respectively, and F1 scores were 0.671, 0.970, and 0.982, respectively. These findings indicate that KG-only maintained high precision when it answered, but its low recall substantially limited overall performance, whereas RAG-only and GraphRAG achieved a more favorable balance between precision and recall.

### 3.5. Inter-Rater Reliability

Overall inter-rater agreement across all three reviewers and all 750 outputs was high, with Fleiss’ κ = 0.854 (95% CI: 0.822–0.885). Method-specific Fleiss’ κ values were 0.747 for KG-only, 0.894 for RAG-only, and 0.936 for GraphRAG. Pairwise Cohen’s κ values ranged from 0.841 to 0.872. These findings indicate consistent reviewer agreement across systems, with the highest agreement observed for GraphRAG (Figure 7).

The sensitivity to consensus criteria was performed. Under the stricter unanimous reviewer agreement criterion, accuracy decreased for all systems: KG-only decreased from 56.0% to 41.6%, RAG-only decreased from 94.8% to 92.4%, and GraphRAG decreased from 96.8% to 95.6%. The largest reduction occurred for KG-only, suggesting that disagreement was mainly driven by short or sparse KG-only responses, while both retrieval-augmented systems remained highly consistent. The relative ranking was unchanged under this stricter rule, with GraphRAG remaining highest.

Fleiss’ κ was used to quantify multi-rater agreement, and pairwise Cohen’s κ was used to quantify agreement between pairs of raters. Bootstrap 95% confidence intervals were estimated using 2000 iterations. Descriptive agreement categories are shown using the Landis–Koch thresholds: 0.00–0.20, slight; 0.21–0.40, fair; 0.41–0.60, moderate; 0.61–0.80, substantial; and 0.81–1.00, almost perfect.

### 3.6. RAGAS Automated Retrieval and Answer-Quality Metrics

Automated RAGAS evaluation (Table 4) demonstrated a consistent performance pattern. The KG-only architecture performed lowest across all three metrics. RAG-only and GraphRAG outperformed standalone graph retrieval by substantial margins, with GraphRAG achieving the highest mean scores on two of the three metrics. Context Precision was the sole exception, where RAG-only achieved 0.778, compared with 0.730 for GraphRAG and 0.625 for KG-only. This reflects the standard precision-recall tradeoff inherent to parallel hybrid retrieval. GraphRAG’s primary retrieval advantage lay in Context Recall, where it achieved 0.851 compared with 0.796 for RAG-only (adjusted *p* < 0.001), confirming that graph-derived relational structures recovered a significantly higher proportion of necessary clinical context.

A Friedman test was conducted on n = 250 matched pairs (same questions evaluated by all three methods). Mean (SD) indicates the mean and standard deviation of metric scores. Kendall’s W = chi2/(n × (k − 1)), k = 3 systems; W ranges: 0–1 (0 = no concordance, 1 = perfect concordance). All *p*-values < 0.001. SD means the standard deviation.

### 3.7. Hallucination and Safety Panel

The six-item binary-normalized hallucination panel produced an internally consistent picture (Table 5). All six-panel metrics showed highly significant overall differences (Friedman chi-square statistics: 103.6–242.9; all *p* < 0.001). Hallucination risk was 0.436 for KG-only, 0.132 for RAG-only, and 0.108 for GraphRAG.

Hybrid KG + RAG retrieval demonstrated the most favorable hallucination profile among the three systems. This corresponded to a 75.2% relative reduction in hallucination risk versus KG-only ([(0.436 − 0.108)/0.436]; bootstrap 95% CI: 70.4–79.8%) and an 18.2% relative reduction versus RAG-only ([(0.132 − 0.108)/0.132]; bootstrap 95% CI: 4.6–32.8%). The wide confidence interval for the RAG-only comparison indicates that the GraphRAG advantage on hallucination over RAG-only, while directionally consistent, carries substantial statistical uncertainty and should be interpreted as suggestive rather than definitive evidence of additional grounding benefit beyond RAG-only.

A Friedman test on matched pairs (n = 250 questions) was conducted. Binary conversion: normalized scores ≥ 0.50 = 1 (present), <0.50 = 0 (absent). Hallucination Risk = 1 − Hallucination-Free Score. Kendall’s W = chi2/(n × (k − 1)), k = 3. All *p* < 0.001.

### 3.8. Retrieval Diagnostics and Response-Behavior Characteristics

Retrieval diagnostics indicated that RAG-only incurred the lowest retrieval overhead (mean latency: 4.59 s; median: 4.57 s), followed by KG-only (mean: 6.67 s; median: 6.47 s), whereas GraphRAG exhibited the greatest retrieval latency (mean: 10.66 s; median: 11.00 s), consistent with its parallel multi-source retrieval and evidence-fusion architecture (Table 6). KG-only retrieved a mean of 13.63 graph facts and 7.81 linked chunks per query. RAG-only retrieved 8.00 chunks per query without graph augmentation. GraphRAG integrated 13.63 graph facts, 11.75 selected graph facts, 7.81 KG-linked chunks, 8.00 base RAG chunks, and 10.00 final merged evidence units on average; graph-guided retrieval was activated in 100% of queries (graph-suppression rate: 0.00), confirming that graph context was consistently incorporated into evidence assembly at every inference step. The RAG-anchor rate of 0.448 indicates that approximately 44.8% of GraphRAG responses were ultimately determined by the monotonic RAG fallback, consistent with the fallback-decomposition analysis reported in Section 3.11. Response-behavior diagnostics further distinguished KG-only from the two retrieval-augmented systems: the abstention rate was 0.8% and the IDK rate was 0.4% for KG-only, compared with 0% for both RAG-only and GraphRAG, reflecting KG-only’s reduced coverage for queries whose answer entity was absent or ambiguously represented in the knowledge graph.

In Table 6, values are reported as means unless otherwise indicated. Retrieval latency reflects total pipeline wall-clock time per query. The graph-guided retrieval rate indicates the proportion of queries for which graph-guided chunks (guided_rag_chunks > 0) were generated. The graph-suppression rate indicates the proportion of queries in which the graph evidence was overridden (ignore_graph_rate). The RAG-anchor rate indicates the proportion of GraphRAG responses determined by the monotonic RAG fallback mechanism. Abstention and IDK rates are reported as proportions. Dashes indicate metrics not applicable to a given method or not explicitly logged in that retrieval configuration.

### 3.9. Response Readability

Response readability was assessed as a linguistic quality dimension of generated text, complementing clinical accuracy and grounding metrics [43]. We measured the Flesch Reading Ease (FRE) and Flesch–Kincaid Grade Level (FKGL) across all 250 matched responses per architecture (Figure 8; Table S1). All three systems produced responses below the patient-education threshold (FRE ≥ 60), indicating that linguistic adaptation would be required before unsupervised patient-facing deployment. KG-only exhibited the lowest FRE (mean: 35.27, SD: 16.99) and highest FKGL (mean: 12.77, SD: 3.11), reflecting the technical phrasing of its compact, graph-derived answers. RAG-only (FRE mean: 40.90; FKGL mean: 12.05) and GraphRAG (FRE mean: 41.32; FKGL mean: 12.01) produced comparable readability scores; both were significantly more readable than KG-only on both metrics (all pairwise adjusted *p* < 0.001; effect sizes r = 0.38–0.50), with no significant difference between them (FRE adjusted *p* = 0.503; FKGL adjusted *p* = 1.000). Response length differed markedly: KG-only produced substantially shorter outputs (mean: 63.09 words) than RAG-only (mean: 134.57 words; adjusted *p* < 0.001) and GraphRAG (mean: 126.75 words; adjusted *p* < 0.001). GraphRAG responses were modestly but significantly shorter than RAG-only (adjusted *p* = 0.043), consistent with its structured graph-guided evidence selection. The brevity of KG-only responses reflects limited evidence retrieval rather than linguistic efficiency; the longer outputs of RAG-only and GraphRAG carry clinically richer content at an acceptable trade-off in reading complexity.

### 3.10. Category-Wise Performance and Emergency-Category Severity Stratification

Category-level analysis showed substantial variation in KG-only performance. KG-only achieved a 0% accuracy only in Diet/Food After Surgery and showed low performance in Nausea/Vomiting, Sexual Activity, and Travel/Drive, each at 20%. These findings suggest that KG-only retrieval struggled most with topics requiring broader narrative clinical guidance rather than isolated graph facts. KG-only performed better in Pain Management (90.0%) and reached an 80.0% accuracy in Alarm Signs, Sutures, and Need for Home Assistance. RAG-only achieved a 100% accuracy in 16 of the 21 clinical/emergency categories, while GraphRAG achieved a 100% accuracy in 18 of 21 categories. Because aggregate completeness scores can obscure topic-specific failure patterns, we also examined mean completeness across individual clinical categories and boundary-condition prompts.

The category-wise completeness analysis provides a more granular view of where each retrieval architecture succeeded or failed (Figure 9). KG-only did not underperform uniformly; rather, its weakest areas were categories that required broader explanation or synthesis across several postoperative instructions, such as diet after surgery, nausea and vomiting, sexual activity, showering, support garments, and travel or driving. This pattern suggests that isolated graph facts were often insufficient when the patient question required contextual guidance rather than a single retrieved relation. By comparison, RAG-only and GraphRAG produced consistently complete responses across most categories, reflecting the value of retrieving fuller narrative context from the postoperative knowledge corpus. GraphRAG had the highest or joint-highest completeness scores in several clinically important areas, including diet after surgery, nausea and vomiting, medication interactions, support garments, sutures, showering/bathing, travel/driving, and several out-of-scope domains. This pattern suggests that the hybrid architecture may be most useful when a response must combine narrative postoperative instructions with structured clinical relationships or safety-boundary decisions. These findings support the broader interpretation that the benefit of retrieval-augmented systems is not limited to binary correctness; they also provide more complete and actionable responses across the range of questions patients are likely to ask during recovery.

Emergency-category severity stratification was performed. The 20 emergency-intent questions were post hoc stratified into high-severity and intermediate-severity presentations. High-severity presentations included immediately life-threatening or potentially limb-, vision-, or organ-threatening scenarios such as severe chest pain, heavy bleeding, sudden dyspnea, suspected anaphylaxis, suspected DVT/PE, sudden neurological deficit, monocular vision loss, high fever with chills, and possible opioid overdose. Intermediate-severity presentations included urgent but less immediately life-threatening concerns such as wound infection signs, drain dislodgement, uncontrolled pain, persistent vomiting, shaking/sweating without a more specific diagnosis, and wound dehiscence without active hemorrhage. Using this stratification, RAG-only and GraphRAG each correctly handled 12 of 14 high-severity emergencies (85.7%, 95% CI: 60.1–96.0%) and 3 of 6 intermediate-severity emergencies (50.0%, 95% CI: 18.8–81.2%). KG-only also correctly handled 12 of 14 high-severity emergencies (85.7%, 95% CI: 60.1–96.0%), but only 1 of 6 intermediate-severity emergencies (16.7%, 95% CI: 3.0–56.4%). The two high-severity emergencies missed by both RAG-only and GraphRAG were a suspected DVT/PE presentation and sudden loss of sensation in an arm, both of which should prompt emergency escalation.

### 3.11. Decomposition of the Monotonic RAG Fallback Contribution

The observed fallback rate of 44.8% raises an important interpretive question: How much of the headline overall accuracy and hallucination risk reported for GraphRAG reflects the hybrid graph architecture, and how much reflects the standard RAG safety fallback? The present design does not permit an experimental answer to this question. However, because the fallback substitutes the RAG-only output verbatim, the observed aggregate can be partitioned analytically under an explicitly stated assumption, which we set out below.

Because the fallback mechanism substitutes the RAG-only output verbatim, the GraphRAG output stream can be partitioned into two strata: the 44.8% of outputs (112 of 250 queries) that were identical to the corresponding RAG-only responses and the complementary 55.2% that were produced without fallback substitution by the hybrid pipeline.

Analytical decomposition of hallucination risk was performed. Let λ denote the proportion of fallback-substituted outputs (λ = 0.448), π_n_ represent the estimated hallucination risk in the native-GraphRAG stratum, and π^RAG^ denote the baseline hallucination risk in the RAG-only stratum (π^RAG^ = 0.132, identical to the standalone RAG-only system). The overall GraphRAG hallucination risk of 0.108 decomposes as follows:

0.108 = (0.448 × 0.132) + (0.552 × π_n_)

Solving for the native-stratum term yields an estimated hallucination risk of 0.089 in the native-hybrid stratum under the stated decomposition assumption. Relative to the RAG-only value of 0.132, this corresponds to a 32.9% lower estimated risk within that stratum, which comprises the 55.2% of outputs not substituted by fallback. The aggregate relative reduction of 18.2% reported in Section 3.7 therefore reflects the complete system, including fallback. This partition follows arithmetically from the assumption that fallback-substituted outputs carry the RAG-only risk exactly; it is not a measurement of any independent effect of the graph component and should not be interpreted as one.

This dual-component design reflects a deliberate clinical safety architecture. Graph augmentation is intended to improve context recall and completeness where it is informative, while the monotonic RAG fallback anchors the system to the validated RAG-only response where graph-derived evidence does not improve the answer. This decomposition should be interpreted as a conditional analytical estimate rather than a substitute for a formal ablation. Future work should evaluate the GraphRAG pipeline with the fallback rule disabled in order to establish empirically whether native graph augmentation confers an independent benefit.

### 3.12. Ecological Validity and the Design Choice of an Expert-Authored Benchmark

The decision to evaluate against an expert-authored 250-question benchmark rather than against authentic patient portal messages was a deliberate one, made to support three goals that are difficult to achieve simultaneously with real patient data: (i) full transparency of the reference answers, which allows automated grounding metrics (RAGAS) to be computed precisely; (ii) systematic coverage of all 28 clinical and out-of-scope categories that a postoperative system must handle, many of which appear rarely or never in any given portal sample; and (iii) the absence of patient privacy and consent constraints that would otherwise prevent the public release of the evaluation set as Supplementary Materials. Closely related work in this space including the postoperative RAG studies and the rare-disease GraphRAG study cited above [26,28] has similarly used expert-authored or curated question sets for these reasons.

Two structural properties of our dataset bear on whether these results are an artifact of expert query phrasing. First, the 30 out-of-scope queries were explicitly drafted in the informal, colloquial register of patient portal messages (e.g., “What is the weather today?”, “Who won the game?”, or “Can you give me stock advice?”); the scope-routing and retrieval components handled these with a safety/refusal accuracy of 93.3% for KG-only, 83.3% for RAG-only, and 93.3% for GraphRAG. Second, the category-level subgroup analysis (Section 3.10) showed that GraphRAG maintained high performance across most clinical categories rather than in only a narrow subset. These observations provide some reassurance that the findings were not driven solely by a narrow subset of expert-authored prompts, but they do not exclude bias associated with benchmark construction. They also do not substitute for a prospective evaluation against authentic patient messages, which remains the critical outstanding empirical question and is discussed in Section 4.10 as a hard limitation of the present work and in Section 4.11 as the primary planned next step.

## 4. Discussion

### 4.1. Principal Findings

This study evaluated three retrieval architectures on a postoperative question-answering task using a blinded review of 750 model outputs by three clinical experts. Reviewers demonstrated almost perfect inter-rater agreement across all evaluated systems (overall Fleiss’ κ = 0.854; 95% CI: 0.822–0.885), confirming the high reproducibility of our human grading schema. We present these clinician ratings as our primary evidence, supplemented by automated grounding metrics (RAGAS and an LLM-as-judge panel) as secondary, converging measures.

Overall accuracy differed significantly across the systems (Cochran’s Q = 188.06, df = 2, *p* < 0.001). Both retrieval-augmented pipelines substantially outperformed standalone knowledge-graph retrieval (adjusted *p* < 0.001 for both McNemar comparisons). The observed overall accuracy difference between RAG-only and GraphRAG was 2.0 percentage points, arising from five discordant question pairs, and did not reach the Bonferroni-adjusted significance threshold (exact McNemar *p* = 0.062; adjusted *p* = 0.188). This comparison is therefore best described as an underpowered and statistically inconclusive signal in the direction of GraphRAG rather than as evidence of superiority over a well-implemented RAG-only system. Subgroup analysis further clarified system behavior. Clinical medical accuracy, calculated across the 200 in-scope postoperative questions and 20 emergency-intent questions, was 50.9% for KG-only, 96.4% for RAG-only, and 97.3% for GraphRAG. Safety/refusal accuracy, calculated across the 30 out-of-scope questions, was 93.3% for KG-only, 83.3% for RAG-only, and 93.3% for GraphRAG; KG-only and GraphRAG were therefore identical on this measure, and the apparent GraphRAG advantage in refusal behavior reported previously does not hold. These findings indicate that both retrieval-augmented systems substantially outperformed standalone KG-only retrieval for clinically relevant postoperative question answering, while GraphRAG showed the highest point estimate for overall and clinical accuracy. The poor performance of KG-only suggests that a basic, corpus-derived knowledge graph alone cannot adequately support patient-facing clinical question answering over narrative postoperative data, likely because it lacks the linguistic flexibility needed to handle natural patient phrasing.

Secondary automated grounding: Our automated evaluations supported the clinical findings, providing clear diagnostic insights into how these different retrieval architectures behave. GraphRAG’s primary mechanical advantage lay in its superior context recall (0.851 versus 0.796 for RAG-only). This metric serves as a critical safety proxy in postoperative care, where omitting a single warning sign or drug instruction can lead to patient harm. By traversing structured relationships, the graph pulled in clinical connections scattered across different narrative text chunks that standard vector searches bypassed. RAG-only maintained a slight edge in context precision (0.778 versus 0.730 for GraphRAG), illustrating the classic breadth-specificity tradeoff of hybrid systems. Clinician-rated performance remained high despite the broader retrieved context. GraphRAG also achieved a higher mean answer-similarity score, consistent with stronger semantic alignment with the reference answers [14,44].

Beyond retrieval, the six-item automated panel characterized the safety and grounding of the final answers. Both retrieval-augmented systems substantially outperformed KG-only across the automated dimensions, while RAG-only and GraphRAG showed broadly similar performance overall. GraphRAG achieved the most favorable values on most dimensions, including Factual Correctness, Faithfulness, Completeness, Hallucination-Free Score, and Hallucination Risk. GraphRAG achieved the highest Hallucination-Free Score and the lowest Hallucination Risk (0.108 versus 0.132 for RAG-only and 0.436 for KG-only). Overall, the automated panel was broadly consistent with the clinician-rated findings in distinguishing both retrieval-augmented systems from KG-only, while differences between RAG-only and GraphRAG were comparatively small.

### 4.2. Why KG-Only Retrieval Underperformed

The false negatives generated by the KG-only system point to a clear failure mode. The necessary clinical facts existed within the graph, but the rigid retrieval mechanism could not bridge the gap between colloquial patient language and structured schemas.

For instance, a patient asking “can I have a bath?” did not match the node labeled shower restrictions, and “my scar is itchy” failed to map to wound pruritus. Synonyms and rule-based mappings resolved some simpler variations, but informal patient queries easily bypassed these hardcoded boundaries.

In contrast, the KG-only system achieved a 93.3% accuracy rate on out-of-scope refusals, identical to GraphRAG (93.3%) and higher than RAG-only (83.3%). This performance was driven by an upstream, rule-based scope classifier rather than the graph query itself. This confirms that scope classification should be engineered as an explicit, upstream filter rather than treated as an emergent property of database retrieval, and it indicates that the refusal advantage is attributable to the shared safety router rather than to any retrieval architecture.

We evaluated a basic, corpus-derived graph without external ontology matching to preserve a controlled, matched-corpus design. Aligning the graph to standardized terminologies like SNOMED CT or UMLS would likely raise this baseline performance. This remains an important area for future optimization.

### 4.3. Incremental Contribution of GraphRAG

As reported in Section 4.1, GraphRAG’s principal mechanical advantage was higher context recall. Relational triples such as [fever] → EXPECTED_AFTER → [wound infection] helped retrieve clinical connections that were scattered across different text chunks, whereas its slightly lower context precision confirms that broader retrieval can also introduce additional noise.

The system triggered its monotonic RAG fallback in 44.8% of queries (112 of 250), meaning that nearly half of the answers GraphRAG presented were identical to the corresponding RAG-only outputs. This design was intended to reduce the risk that the hybrid system would perform worse than the vector baseline when graph-derived evidence was sparse or unhelpful. A direct consequence is that the aggregate GraphRAG results reported here characterize the performance of the complete hybrid system and cannot be interpreted as the independent effect of graph augmentation. Because no dedicated ablation experiments were performed with the fallback disabled, graph-guided retrieval removed, or retrieval budgets matched, the contribution of the knowledge-graph component cannot be isolated experimentally from the other elements of the hybrid pipeline. The decomposition presented below should therefore be interpreted as a conditional analytical estimate under a stated assumption rather than as a measured component-level effect.

We used mathematical decomposition to estimate performance within the native hybrid stratum without fallback. Let λ represent the fallback rate (0.448), π_n_ represent native GraphRAG hallucination risk, and π^RAG^ represent RAG-only hallucination risk (0.132). The aggregate risk of 0.108 decomposes as follows.

Within the native-hybrid stratum, the estimated hallucination risk was approximately 0.089, compared with 0.132 for RAG-only, a 32.9% relative difference confined to that stratum. The aggregate relative reduction of 18.2% reported in Section 3.7 reflects the complete system with fallback included. As noted above, this partition follows arithmetically from the decomposition assumption stated in Section 3.11 and is not a measured component-level effect.

This dual-component design serves two clear roles. Graph retrieval improves context recall and factual completeness when the query successfully matches the structured clinical network. When it does not, the monotonic fallback acts as a clinical safety anchor, reverting to the well-validated RAG baseline rather than risking an incomplete answer or unnecessary abstention.

### 4.4. Inter-Rater Reliability and Consensus Sensitivity

The overall Fleiss’ κ of 0.854 indicates robust agreement among the clinicians. This consistency was uniform across pairings, and pairwise Cohen’s κ values ranged from 0.841 to 0.872.

Rater agreement on GraphRAG outputs was high (Fleiss’ κ = 0.936; 95% CI: 0.878–0.979), and agreement was also substantial for KG-only (Fleiss’ κ = 0.747; 95% CI: 0.686–0.803) and RAG-only (Fleiss’ κ = 0.894; 95% CI: 0.829–0.948). KG-only produced short, simple outputs that were easy to classify. GraphRAG produced longer, more detailed responses that combined structured facts with narrative context, generating more borderline cases for clinical evaluation.

This is supported by our sensitivity analysis. Requiring unanimous (all-three-rater) agreement reduced absolute accuracy across all systems while keeping their relative rankings. Accuracy dropped by 14.4 percentage points for KG-only, but only by 2.4 percentage points for GraphRAG. This shows that clinical reviewers agreed consistently on the evaluation of retrieval-augmented answers, whereas brief standalone graph outputs led to more borderline classifications.

### 4.5. Human Evaluation and Automated Grounding as Complementary Measures

Human clinical ratings and automated grounding metrics measure different aspects of performance. Clinicians evaluated clinical accuracy, relevance, and completeness. RAGAS metrics focused on technical grounding and the direct relationship between retrieved context and the generated response.

An output can be clinically safe and useful even if the underlying retrieval does not fully support every statement. Rather than a contradiction, this distinction shows that combining human expert judgment with automated retrieval diagnostics provides a more complete view of clinical system safety than either metric alone.

### 4.6. Relationship to Prior GraphRAG Work

Our findings align with medical evaluations of hybrid retrieval systems. Song and colleagues reported that combining graph and vector retrieval improved diagnostic coverage by over 25 percentage points in rare genetic disease QA, while noting that vector search outperformed standalone graph queries [26]. Similarly, Liu and colleagues demonstrated high sensitivity and specificity in triaging patient portal messages using a knowledge-graph-based RAG pipeline with hierarchical community summarization [27]. Our study adds to this literature by conducting a controlled, three-arm evaluation (KG-only versus RAG-only versus GraphRAG) on an identical clinical corpus, comparing structured and unstructured retrieval under matched conditions.

We did not compare our pipeline directly with the community-detection-based GraphRAG architecture proposed by Microsoft [24]. Their system focuses on query-focused summarization across large, unstructured datasets, whereas our clinical pipeline is optimized for single-question retrieval against targeted patient guidance. Evaluating these two approaches side-by-side remains an important area for future research.

The percentage-point clinical accuracy gap between standalone knowledge graph and the retrieval-augmented systems is likely due to the rule-based, non-ontology-aligned nature of our graph rather than a fundamental limitation of graph structures. A more detailed clinical graph aligned with unified ontologies like SNOMED CT or UMLS [34,45] would likely improve the performance of standalone graph retrieval. Additionally, the modest, non-significant difference in human-rated overall accuracy between RAG-only and GraphRAG suggests that, when working with a well-curated corpus and carefully engineered prompts, different retrieval methods may achieve similar clinical utility. In this clinical context, implementing some form of retrieval is far more important than which specific retrieval architecture is selected.

Prior GraphRAG studies have tended to emphasize downstream answer quality, coverage, and consistency more than operational retrieval behavior [26,27]. The present study additionally reports retrieval diagnostics including latency, evidence composition, graph-suppression rate, and abstention behavior to characterize architectural differences at the system level, addressing a reporting gap in the current clinical GraphRAG literature. Notably, no prior clinical GraphRAG study has evaluated response readability alongside accuracy and safety, despite evidence that AI-generated clinical outputs must meet communicative as well as factual standards to be clinically actionable [14,46]. All three architectures produced responses above the recommended 8th-grade reading level (FKGL range 12.01–12.77) and below the patient-education FRE target of 60, a finding with direct implications for unsupervised patient-facing deployment and one that motivates future work on plain-language generation constraints.

### 4.7. Clinical Implications and Emergency-Category Caveat

For surgical departments designing patient-facing clinical tools, our findings suggest three main considerations: First, rule-based, corpus-derived knowledge graphs operating alone are not sufficient for patient-facing question answering. Whether this limitation applies to formal, ontology-aligned clinical graphs remains an open question. Second, for clinical settings where grounding and completeness are the primary requirements, hybrid graph-retrieval is a strong candidate architecture. However, for applications where system latency, engineering simplicity, or ease of maintenance are more important, a well-tuned RAG-only architecture may be sufficient. Third, patient-facing clinical applications must prioritize output faithfulness alongside clinical accuracy. Patients acting on AI-generated instructions without clinical review cannot easily identify or correct unsupported claims.

Regarding emergency triage, special safety precautions are necessary. GraphRAG achieved a 75.0% accuracy on the emergency-intent queries under majority consensus scoring. Post-hoc stratification by clinical severity shows that GraphRAG correctly managed 12 of 14 high-severity, life-threatening presentations (85.7%, 95% CI: 60.1–96.0%). The two missed cases were highly critical: one suggestive of deep vein thrombosis or pulmonary embolism (a unilaterally swollen, hot, painful leg) and one involving a sudden loss of sensation in an arm. For both presentations, the only safe response is immediate referral to emergency services. On intermediate-severity cases (such as localized wound infection or drain dislodgement), GraphRAG’s performance was lower (three of six correct, 50.0%, 95% CI: 18.8–81.2%). These situations require prompt contact with the surgical team rather than emergency room visits [47,48].

These safety gaps lead to four clear engineering requirements. First, upstream classifiers must use strict, rule-based keyword matching to route critical symptoms (such as unilateral limb swelling or sudden neurological deficits) directly to emergency services, bypassing the retrieval-generation loop entirely. Second, any query containing potential emergency indicators must require human clinical review before the response reaches a patient. Third, these systems should not be deployed for autonomous emergency triage until prospective evaluations using dedicated emergency-classification metrics demonstrate sufficiently high sensitivity for critical presentations. Fourth, and most importantly, because two life-threatening presentations were missed and accuracy on intermediate-severity cases was only 50.0%, the architecture evaluated in this study is appropriate only for supervised clinical use in which a qualified clinician reviews every output before it is seen by a patient. It is not suitable for autonomous patient-facing triage in its current form, and we make no claim of readiness for that use.

### 4.8. Deployment Value Proposition and Integration Scenarios

Our proposed GraphRAG architecture is designed to support supervised clinical workflows rather than to operate as an autonomous assistant. We outline three potential integration use cases.

#### 4.8.1. Use Case 1: Asynchronous Patient Portal Triage with Clinician Review

Surgical clinics face high volumes of postoperative patient portal messages. The GraphRAG system can draft initial, source-attributed responses to routine queries. Clinicians can then review, edit and approve these drafts before sending them to patients. By providing explicit citations to the clinical source corpus, the system supports quick clinical verification, reducing drafting time while keeping the clinician in the loop [5,6,30].

#### 4.8.2. Use Case 2: Augmented Nurse-Triage Decision Support

After-hours patient calls are often managed by nurse triage lines. GraphRAG can assist triage staff by quickly retrieving relevant sections of surgical protocols and drafting clinical guidance. The clinician or nurse can then confirm, modify or escalate the response. The rule-based upstream classifier provides an additional safety layer by routing emergency queries directly to standard emergency protocols.

#### 4.8.3. Use Case 3: Patient-Education Content Drafting

Surgical teams can use the system in an offline, non-interactive mode to draft postoperative patient education materials. Because content generation occurs offline, the latency difference between GraphRAG and RAG-only (10.66 s versus 4.59 s) is not a limiting factor. This workflow allows clinics to leverage the hybrid system’s recall and completeness advantages under full clinical review before publication.

This system is not designed for unsupervised, real-time communication with patients. Its incomplete emergency-intent performance and the lack of prospective real-world validation mean it should not be deployed in any setting where a missed critical symptom could lead to patient harm. Within supervised workflows, however, it can help reduce documentation workloads, accelerate response times for routine queries and provide consistent, source-attributed clinical content drawn from the institution’s own knowledge corpus.

Three factors should guide any clinical deployment. The first factor is retrieval latency: GraphRAG’s 10.66-s average latency is suitable for asynchronous drafting but may feel slow for real-time conversational tools. RAG-only (4.59 s) is better suited for interactive applications. The second factor is knowledge base maintenance: The system’s accuracy depends on the quality of its underlying clinical documents. Organizations must establish clear processes for updating and versioning their clinical protocols. The third factor is continuous auditing: deployments should include active monitoring of system refusal rates, abstention rates, and clinician modification rates to catch and address performance drift over time.

### 4.9. Novelty Positioning

This study makes five contributions that distinguish it from prior clinical GraphRAG evaluations, collectively constituting a controlled architectural benchmark rather than a single-system demonstration.

First, the study focuses on the application to patient-facing postoperative guidance. Prior clinical LLM evaluations have concentrated on diagnostic triage, emergency classification, and clinical note summarization [8,15,16]. Postoperative guidance represents a structurally harder retrieval task: Safe responses must simultaneously integrate procedure-specific recovery timelines, wound-care instructions, medication protocols, complication thresholds, and emergency escalation criteria—a multi-facet retrieval demand that single-hop vector search may miss when relevant facts are distributed across logically related but lexically dissimilar document segments. This application context motivated the hybrid graph-plus-vector architecture and provided the ecologically valid testbed in which its advantage was measured.

Second, a matched three-arm experimental design was employed. To our knowledge, this is the first study to compare KG-only, RAG-only, and GraphRAG under a fully controlled matched design identical source corpus, backbone language model (Gemini 2.5 Flash), embedding model, prompt policy, and blinded clinical-review framework. Prior work has evaluated graph-augmented systems in isolation [25,27] or against parametric-only baselines [15,16], making it impossible to partition the performance advantage of structured graph retrieval from dense vector retrieval. Our design directly compares these architectures under matched conditions but does not experimentally isolate the graph component from the other elements of the hybrid pipeline: RAG-only achieved a 94.8% overall accuracy versus KG-only’s 56.0%, and GraphRAG achieved a 96.8%, providing a head-to-head comparison of dense retrieval and the complete hybrid GraphRAG system in a clinical domain.

Third, the study incorporated an embedded clinical safety architecture with reported outcomes. The GraphRAG pipeline incorporated five explicit safety mechanisms: emergency intent routing, out-of-scope refusal, reflective repair, graph suppression when retrieved graph evidence was sparse, and monotonic RAG fallback. Critically, these mechanisms were not treated solely as implementation details; their activation and associated system behavior were reported. The monotonic RAG fallback was activated in 44.8% of queries (112 of 250; the RAG-anchor rate), quantifying how often the final output reverted to the dense-retrieval baseline, and the graph-suppression rate was 0.00, indicating that graph evidence was incorporated into evidence assembly at every inference step. Safety/refusal accuracy reached 93.3% for GraphRAG across the 30 out-of-scope queries, compared with 83.3% for RAG-only, indicating that the safety framework produced measurably different outcomes rather than uniform template responses across architectures.

Fourth, an analytical decomposition of hybrid system performance was provided. A known limitation of hybrid retrieval benchmarks is that reported performance conflates the contribution of each retrieval stratum. We therefore partitioned GraphRAG outputs into fallback-substituted outputs (final output identical to RAG-only) and native-hybrid outputs (no fallback substitution), and reported the estimated hallucination risk within each stratum under an explicitly stated assumption (Section 3.11). This makes explicit how much of the aggregate result is carried by the RAG-only baseline, rather than attributing the full system-level performance to graph evidence, and provides a reporting template for other hybrid retrieval architectures. We emphasize that this is an analytical partition and not an ablation: it does not establish an independent effect of the graph component.

Fifth, we evaluated patient-facing readability alongside clinical accuracy and safety. Because postoperative guidance is intended directly for patients rather than clinicians, its real-world utility depends not only on factual correctness but also on whether responses are understandable and actionable. We therefore evaluated readability using the Flesch Reading Ease (FRE) and Flesch–Kincaid Grade Level (FKGL) across all 750 system outputs. This analysis adds a crucial patient-centered communication dimension that is often absent from technical GraphRAG evaluations, helping to distinguish algorithmic grounding performance from real-world clinical usability and directly informing safe deployment decisions in patients with varying health-literacy needs.

Together, these five features position the present study as a controlled architectural benchmark for retrieval system selection in postoperative clinical question answering. The findings provide evidence relevant to clinical AI deployment teams when deciding between knowledge-graph, dense-retrieval, and hybrid architectures: under the conditions tested, GraphRAG’s incremental accuracy difference over RAG-only was modest (2.0 percentage points) and not statistically significant after correction. The hybrid system also showed a lower point estimate for hallucination risk, although the difference from RAG-only was not statistically significant. These potential benefits therefore need to be weighed against the additional architectural complexity and latency.

### 4.10. Limitations

Our findings should be interpreted in light of several limitations. The evaluation set was composed of expert-authored questions rather than authentic patient messages. Although the curated benchmark enabled clear reference answers, balanced coverage across clinical categories, and a shareable evaluation resource, real patient messages often include typographical errors, ambiguity, and multiple concurrent concerns. Evaluation using real patient portal data is therefore an important next step. A 50-question calibration subset was used during hyperparameter selection and remained within the final 250-question aggregate analysis. Although parameters were fixed before final scoring, this approach may modestly overestimate performance compared with evaluation on a fully independent held-out dataset. Future studies should assess the fixed pipeline using an external benchmark. The benchmark questions were authored for this study, and the detailed authoring workflow, including any difficulty grading, independent item-level re-review, and formal de-duplication screening, was not prospectively documented; future benchmark releases should record this provenance explicitly.

The observed performance of GraphRAG reflects a multi-component hybrid design that includes query expansion, a second retrieval stage, evidence fusion, and a monotonic RAG fallback. This configuration represents the intended hybrid system, but it also means that the study does not isolate the contribution of the knowledge graph itself. No dedicated ablation experiments were performed with the fallback disabled, with graph-guided retrieval removed, or against a RAG-only baseline matched for retrieval and evidence budget. Accordingly, the contribution of the knowledge-graph component cannot be isolated experimentally from the other elements of the hybrid pipeline. The analytical decomposition reported in Section 3.11 is a partition of the observed aggregate under a stated assumption rather than an experimental isolation, and the estimated native-hybrid hallucination risk of approximately 0.089 should therefore be interpreted as conditional on that assumption. These ablations constitute an important priority for future work and a principal limitation of the present study. This study was also not designed as a superiority trial: The RAG-only versus GraphRAG comparison did not reach the Bonferroni-adjusted significance threshold (adjusted *p* = 0.188), rested on only five discordant question pairs, and produced a wide odds-ratio interval that includes the null value. The corresponding difference in estimated hallucination risk was likewise not statistically significant (adjusted *p* = 0.771, negligible effect size). Neither result should be interpreted as evidence of broad superiority over a well-implemented RAG-only system. Finally, latency was measured, but detailed API-call counts, token usage, monetary cost, graph-processing cost, and cost per correctly answered question were not prospectively logged. Therefore, the full computational and financial overhead of GraphRAG relative to its small, non-significant accuracy difference compared with RAG-only could not be quantified.

The evaluation was limited to a single surgical knowledge base in plastic and reconstructive surgery, and performance may not generalize to specialties with different clinical workflows and postoperative protocols. In addition, the knowledge graph was generated through rule-based extraction and was not aligned with external clinical ontologies. Incorporating standardized resources such as SNOMED CT or UMLS may improve standalone graph retrieval and representation quality [34,45]. Both the graph and retrieval components were implemented using lightweight local artifacts rather than production-grade graph and vector databases. This design reduced infrastructure-related complexity and enabled a controlled comparison focused on retrieval strategy rather than database implementation. However, it limits conclusions about scalability, indexing efficiency, latency, deployment robustness, and performance under larger clinical workloads. Future work should repeat the three-arm comparison using scalable graph and vector database systems.

All evaluations used Gemini 2.5 Flash, and findings may differ with other commercial or open-source language models. The hallucination and safety panels also used Gemini 2.5 Flash as an LLM judge, which introduces the possibility of model self-preference bias. We sought to reduce this risk by anchoring assessments to expert reference answers, evaluating outputs in a unified framework, and using predefined scoring rubrics. Human reviewer ratings, which were independent of the LLM judge, remain the primary outcome. Standalone graph responses were shorter on average than RAG-only and GraphRAG responses (63 versus 135 and 127 words, respectively). Although reviewers were blinded to method labels, the shorter graph-only answers may have been recognizable and could have introduced evaluation bias. This concern is less relevant to the RAG-only versus GraphRAG comparison, where response lengths were nearly identical.

Finally, 50 of the 250 benchmark questions involved emergency-intent or out-of-scope prompts that required safety-boundary handling rather than routine postoperative guidance. This composition should be considered when interpreting overall accuracy. We also did not assess performance across demographic groups, health-literacy levels, or languages, all of which are necessary considerations before clinical translation. In addition, the evaluated system was a hybrid KG + RAG framework rather than Microsoft’s hierarchical GraphRAG architecture.

### 4.11. Future Directions

Our immediate next step is to evaluate the system against real, de-identified patient messages under appropriate ethical approval. Testing across other surgical specialties, such as orthopedics or surgical oncology, will help determine if the hybrid retrieval advantages are generalizable.

On the technical side, adding a reranking step to score retrieved knowledge graph triples before generation could improve context precision without reducing recall. We also plan to investigate more detailed graph construction methods using clinical NLP, formal clinical ontologies, and adaptive retrieval routing that automatically selects precision-focused or recall-focused settings based on query type.

Most importantly, the clinical utility of these systems must be validated through prospective clinical trials. These studies should measure patient-centered outcomes, including information recall after discharge, adherence to care plans, correct recognition of safety symptoms, and impacts on avoidable emergency department visits [49,50,51].

### 4.12. Evidence Required for Clinical Translation

While our benchmark results are promising, they are not sufficient to support clinical deployment. We outline several categories of evidence required before these architectures can be safely introduced into patient care [46,49,52].

First, we must test these models prospectively on real, de-identified patient portal messages. Real-world messages often contain grammatical errors, medical jargon, and multiple overlapping questions, which can affect retrieval performance.

Second, we need to conduct multi-specialty and multi-site validations. This requires evaluating the system across different healthcare centers and surgical specialties to ensure the retrieval architecture generalizes well to different clinical documentation styles.

Third, patient-centered clinical trials are necessary. We must evaluate whether deploying this tool improves patient outcomes, such as safety-symptom recognition, adherence to care plans, and reducing unnecessary emergency department visits.

Fourth, workflow integration and human-factors studies must be performed. We must evaluate how these tools fit into clinical practice, assessing whether drafting tools reduce clinician workloads or if editing drafted responses adds new administrative burdens.

Fifth, demographic and equity audits are required. We must conduct formal subgroup analyses to ensure the system performs consistently across different patient demographics, literacy levels, and languages [50,53].

Sixth, automated grounding evaluations should be validated across cross-family model configurations. These assessments should use independent, alternative language models to ensure results are free from model-specific scoring biases.

Finally, any clinical implementation requires long-term monitoring and drift detection. This involves establishing continuous monitoring frameworks to track system refusal rates, response drift, and clinical accuracy as medical protocols and model APIs change over time. Until these categories are addressed, the findings should be interpreted as evidence of benchmark performance and architectural feasibility, not as evidence that the system is ready for autonomous patient-facing clinical use.

## 5. Conclusions

In this controlled three-arm benchmark comparison, blinded clinical-expert evaluation showed that both retrieval-augmented systems substantially outperformed standalone knowledge-graph retrieval, with overall accuracies of 96.8% for GraphRAG, 94.8% for RAG-only, and 56.0% for KG-only. Inter-rater agreement was high (Fleiss’ kappa = 0.854), supporting the consistency of the human evaluation. The pairwise comparison between RAG-only and GraphRAG did not reach the Bonferroni-adjusted significance threshold (exact McNemar *p* = 0.062; adjusted *p* = 0.188); the absolute difference was 2.0 percentage points and rested on five discordant question pairs. Because the monotonic RAG fallback determined 44.8% of GraphRAG outputs, and because no component ablations were performed, these results characterize the complete hybrid system rather than an independent benefit of graph augmentation.

A reasonable interpretation of these findings is that retrieval augmentation is essential for postoperative question answering, while the incremental benefit of graph augmentation over a well-implemented RAG-only system is more limited and context-dependent. Given a curated knowledge corpus, careful prompt design, and robust retrieval tuning, RAG-only may be sufficient for many lower-complexity deployment settings. GraphRAG may be most useful when faithfulness, completeness, and evidence traceability are primary design priorities, particularly for complex or safety-sensitive postoperative queries.

The present study should be understood as benchmark-stage evidence that informs architectural choice for postoperative clinical question answering, not as evidence of clinical readiness. Before patient-facing deployment, the systems evaluated here require prospective testing on authentic patient messages, external multi-site validation, patient-outcome evaluation, workflow-integration studies, demographic-subgroup analysis, cross-family judge validation, and longitudinal monitoring.

## Figures and Tables

**Figure 1:**
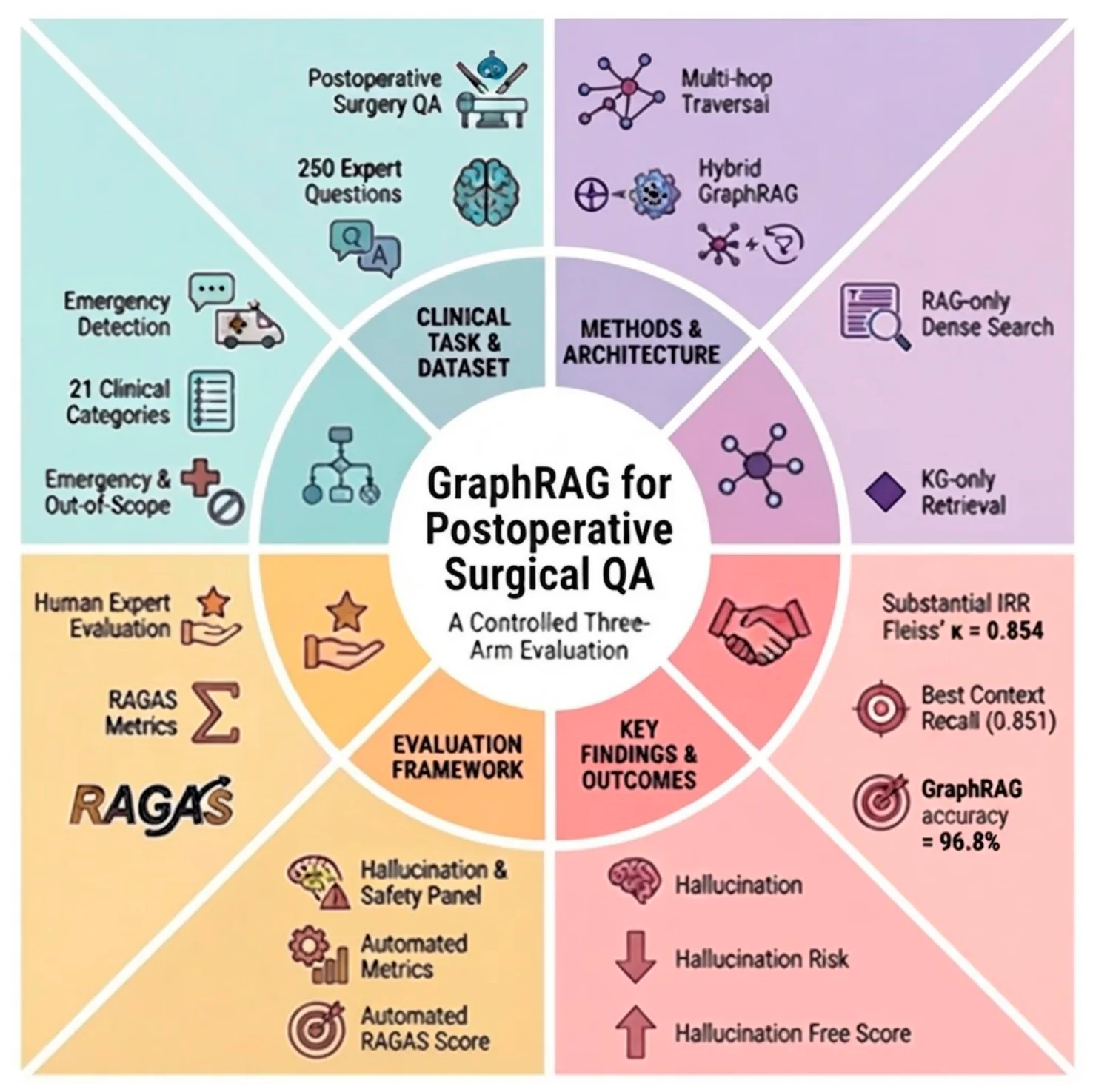
Overview of our proposed methodology for postoperative question answering.

**Figure 2:**
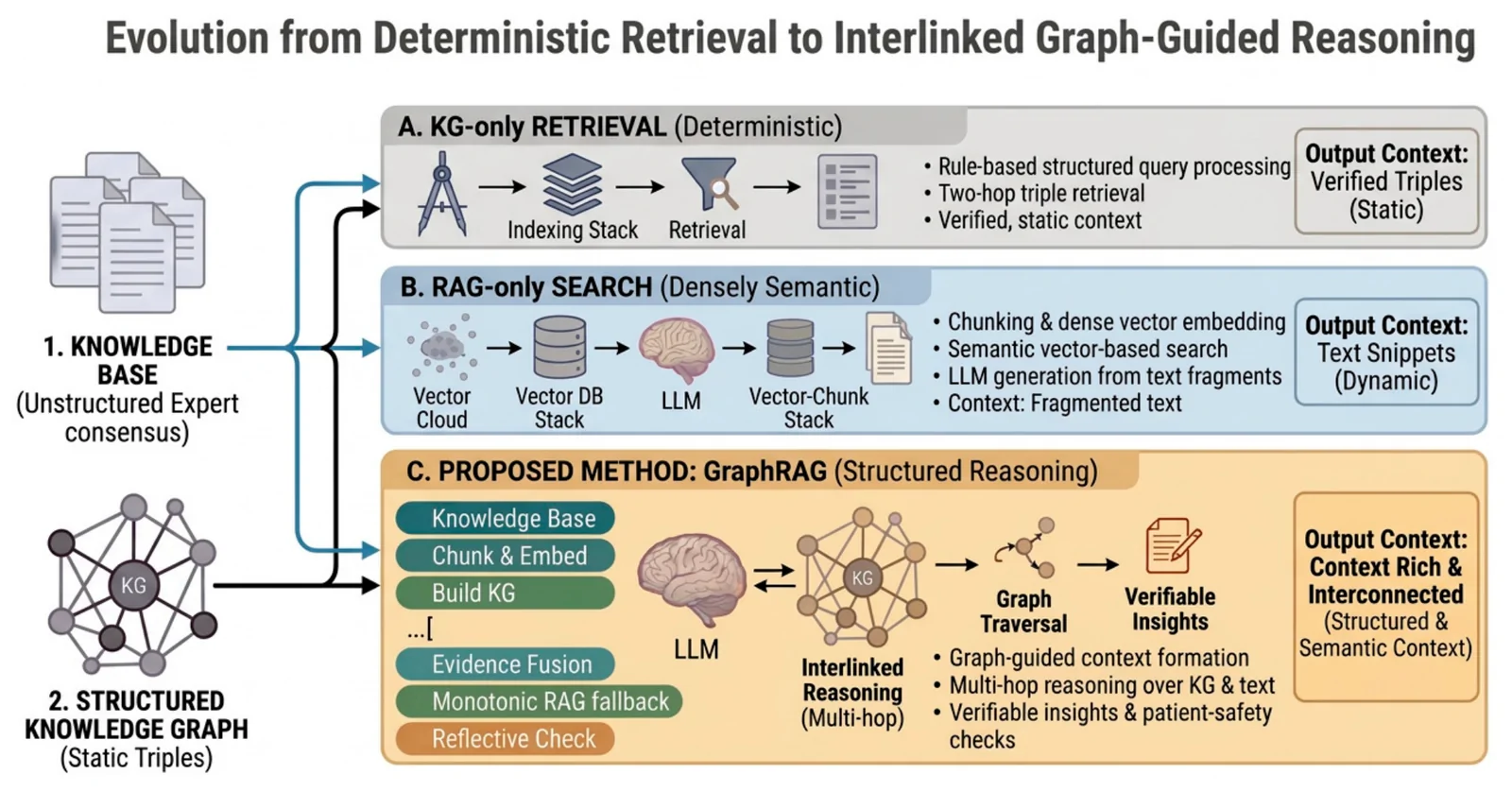
Comparative methodology of clinical QA context formation. Arrows indicate the direction of information flow through each retrieval and generation pipeline. Symbols and icons represent the unstructured knowledge base, vector database, language model, structured knowledge graph, graph traversal, and final output context for the KG-only, RAG-only, and GraphRAG architectures.

**Figure 3:**
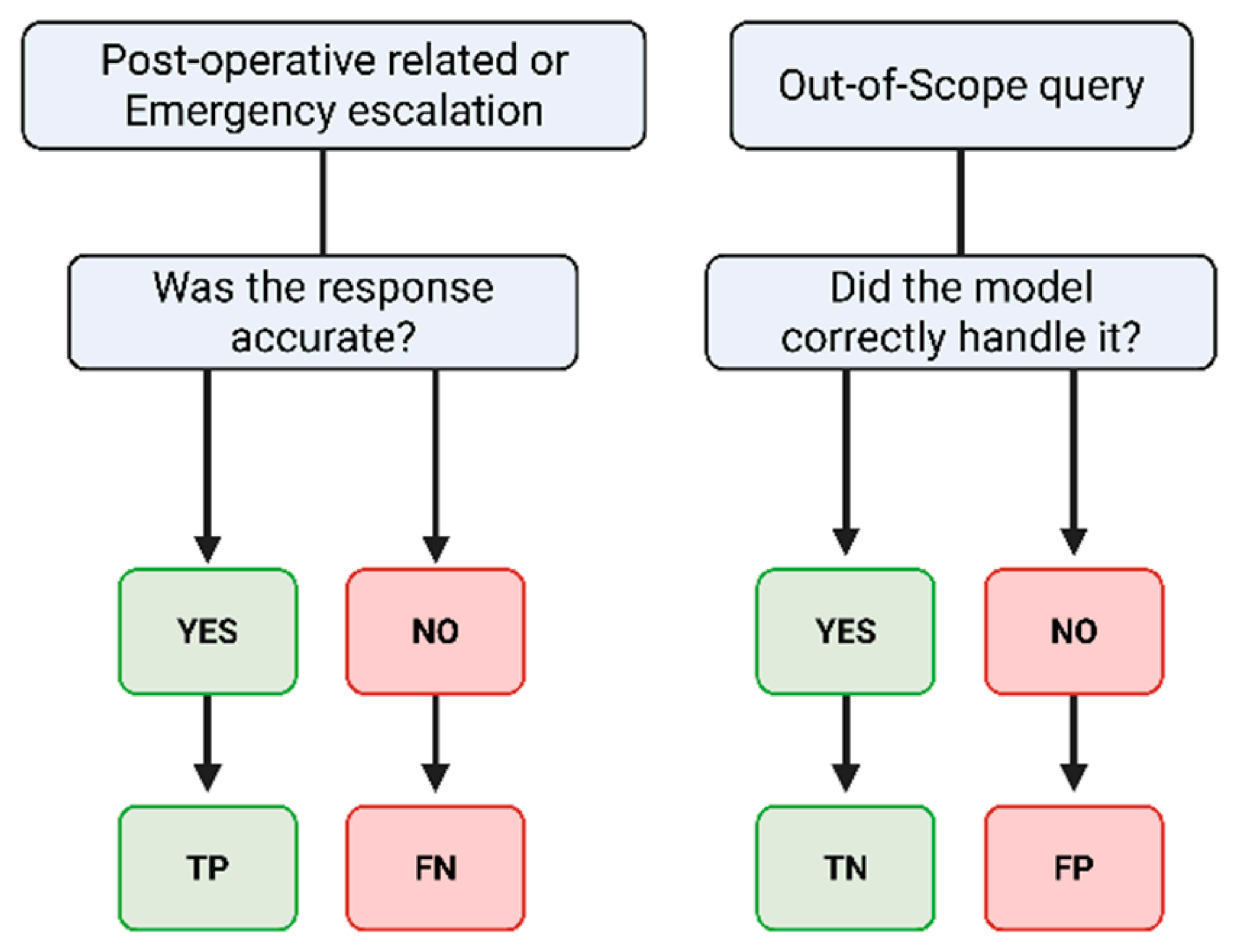
Overall accuracy classification flow diagram. Arrows indicate the direction of the classification decision process from query type and response assessment to the corresponding true-positive, false-negative, true-negative, or false-positive classification.

**Figure 4:**
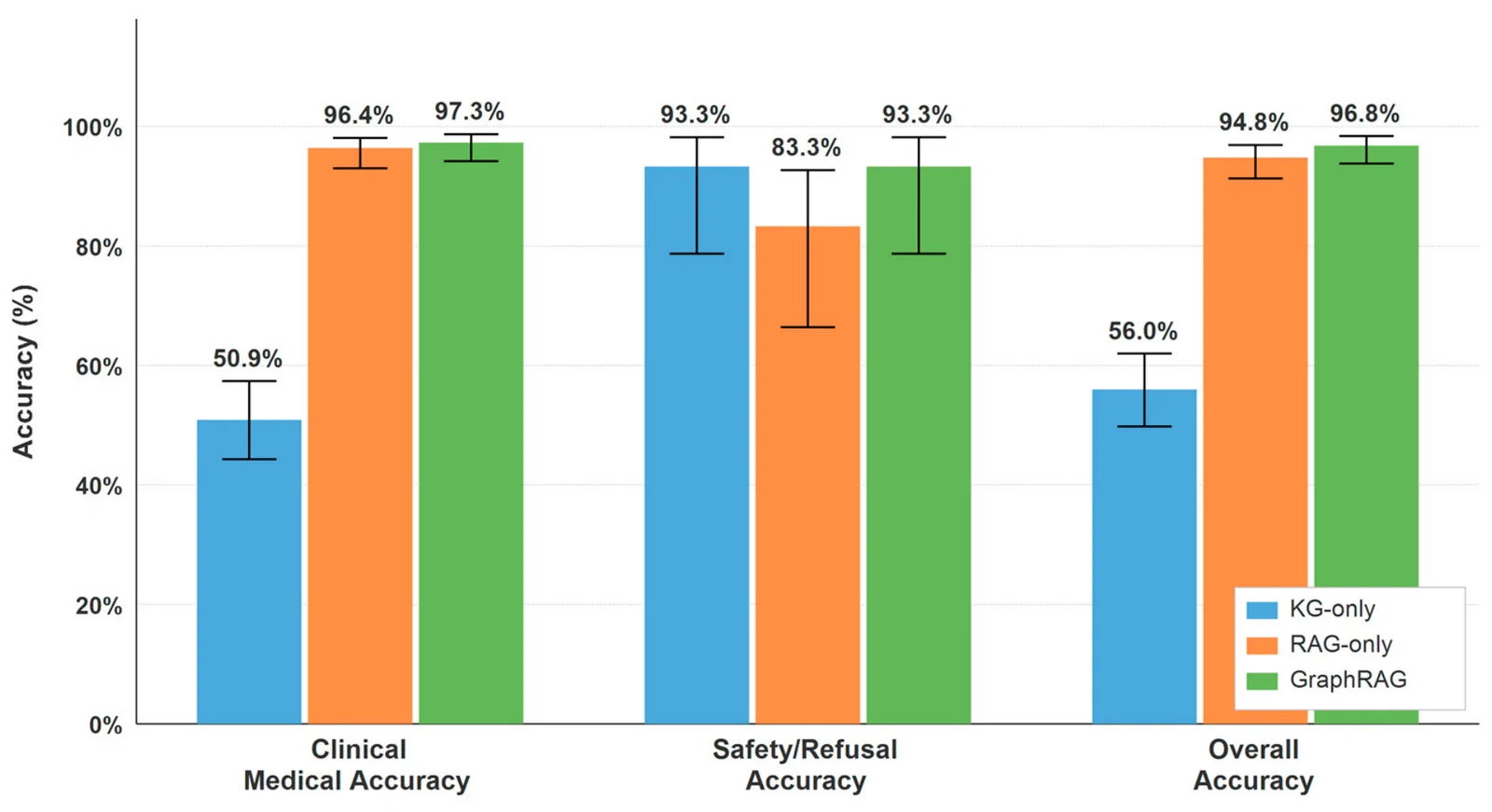
Human-rated accuracy across KG-only, RAG-only, and GraphRAG. Clinical medical accuracy was calculated across 220 clinical/emergency questions, safety/refusal accuracy was calculated across 30 out-of-scope questions, and overall accuracy was calculated across the full 250-question benchmark. Error bars represent 95% Wilson confidence intervals.

**Figure 5:**
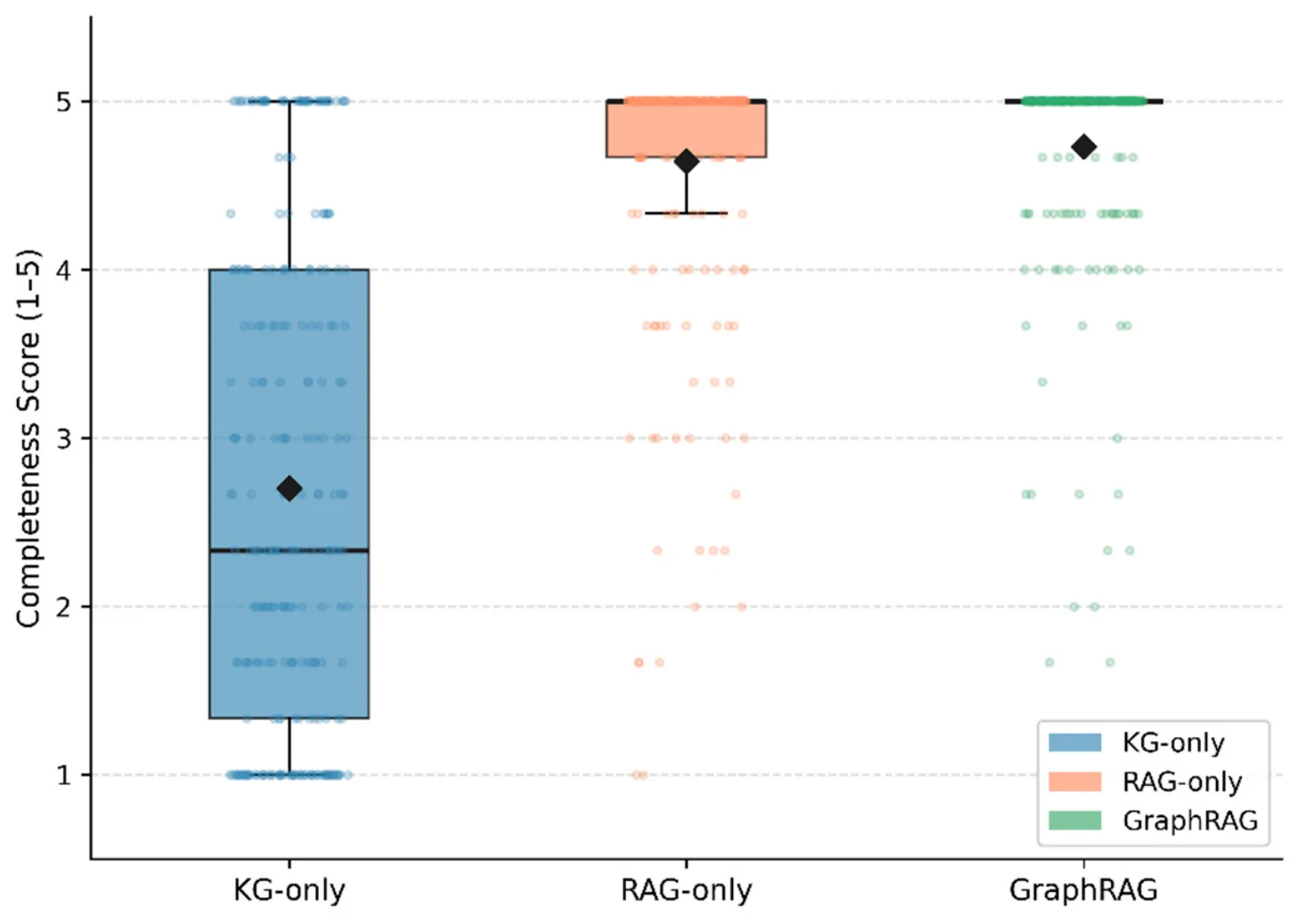
Distribution of clinician-rated completeness scores (1–5 Likert scale, mean of three blinded raters) across KG-only, RAG-only, and GraphRAG (n = 250); the diamond indicates the mean; the horizontal line indicate the median; the boxes indicate the IQRs; the whiskers indicate 1.5 × IQR; the circles indicate individual data points.

**Figure 6:**
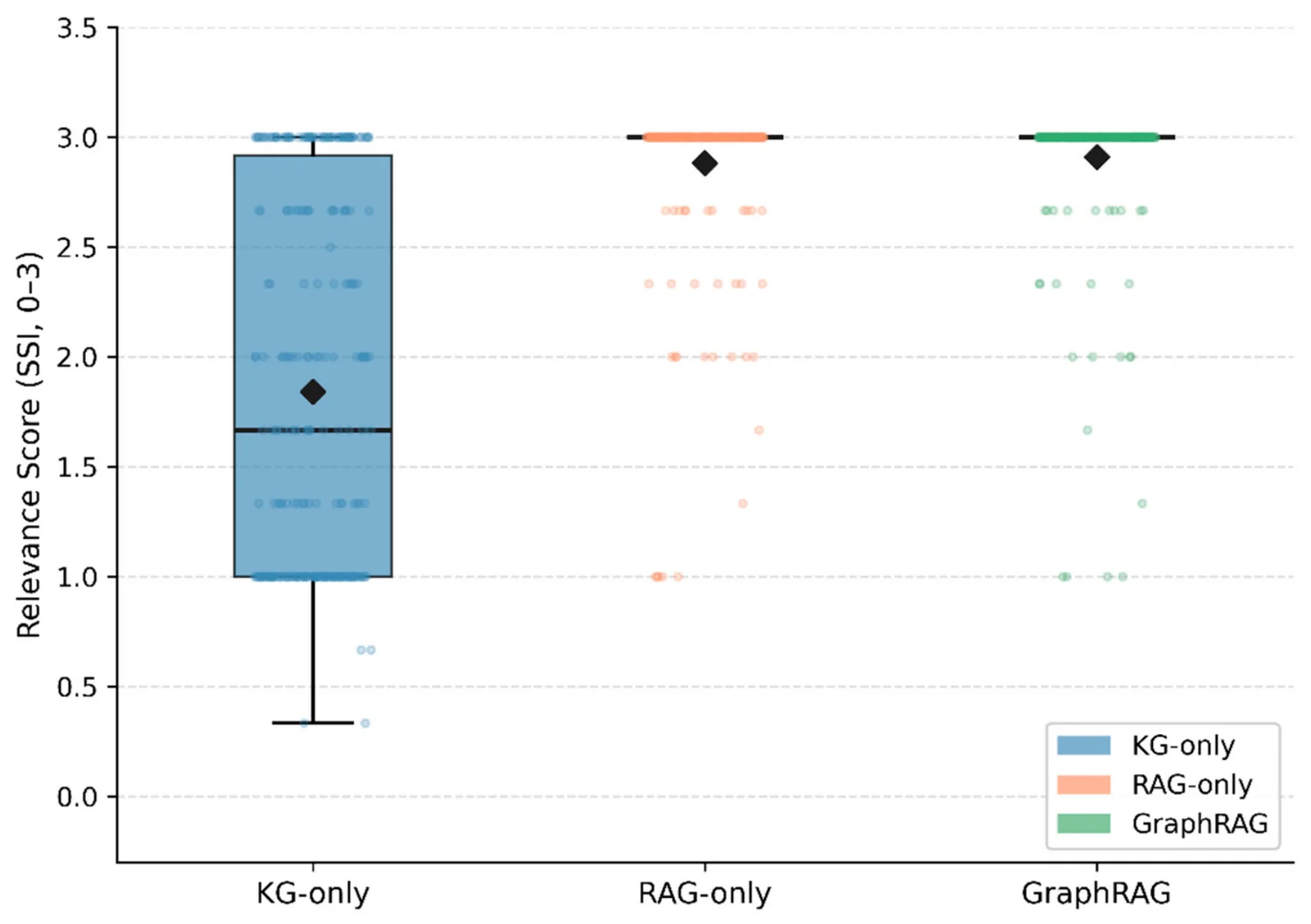
Distribution of clinician-rated relevance scores (SSI index, 0–3, mean of three blinded raters) across KG-only, RAG-only, and GraphRAG (n = 250); the diamond indicate the mean; the horizontal line indicate the median; the boxes indicate the IQRs; the whiskers indicate 1.5 × IQR; the circles indicate individual data points.

**Figure 7:**
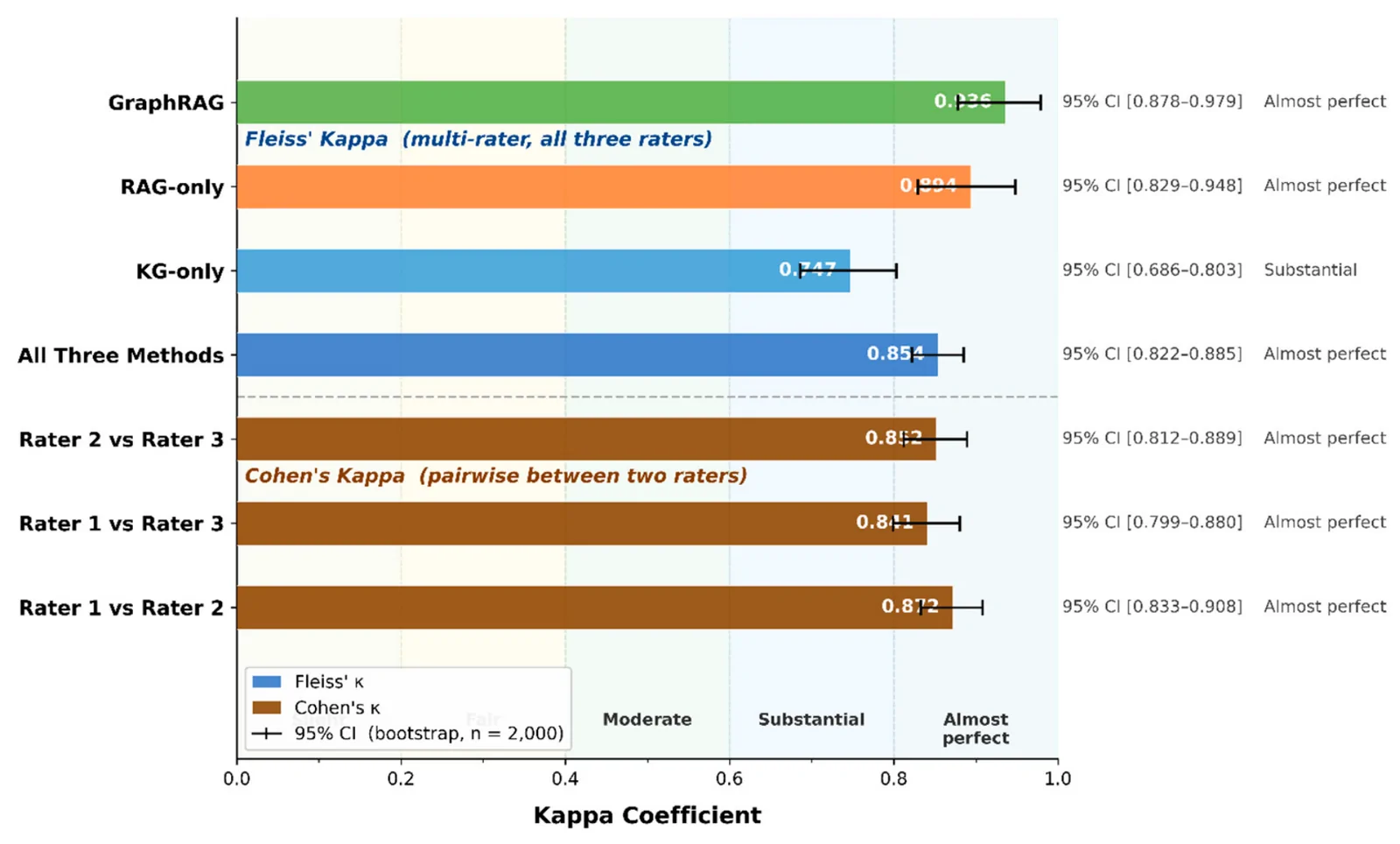
Inter-rater reliability across reviewers and systems.

**Figure 8:**
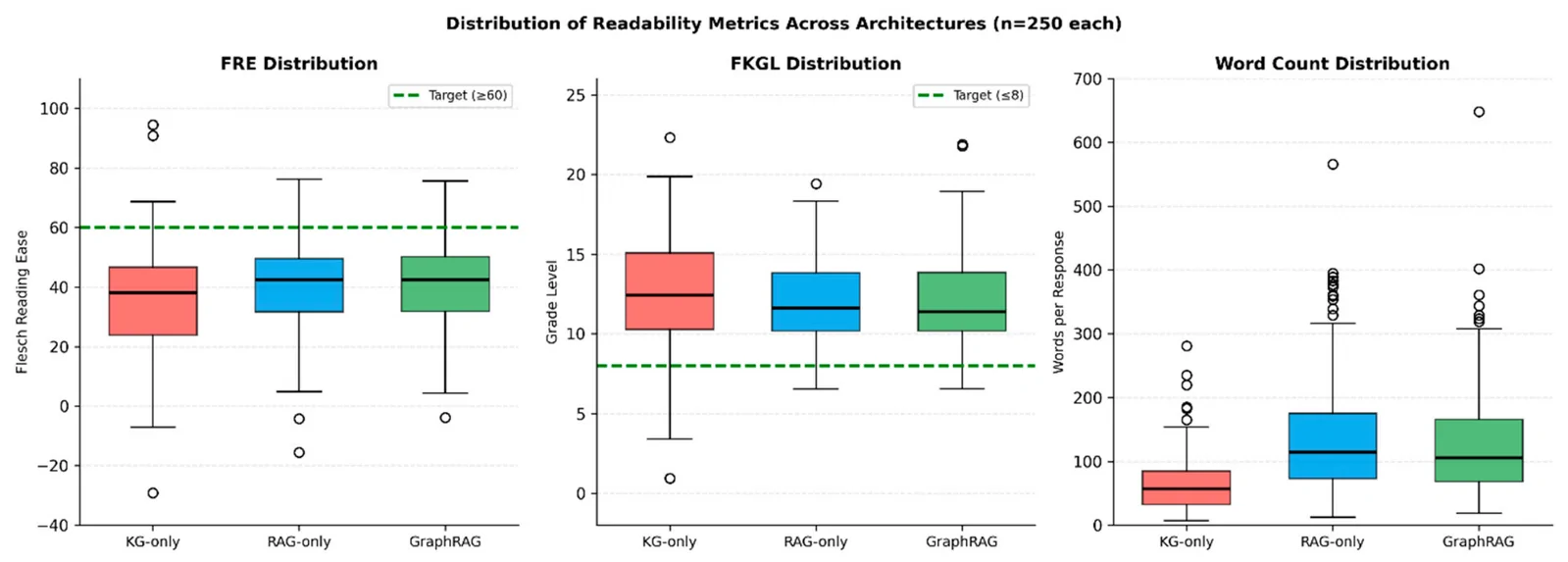
Response readability distributions across KG-only, RAG-only, and GraphRAG (n = 250 per system). Box plots show Flesch Reading Ease (FRE; (**left**)), Flesch-Kincaid Grade Level (FKGL; middle), and word count (**right**). Dashed green lines indicate patient-education targets (FRE ≥ 60; FKGL ≤ 8th grade). All systems fell below the FRE target and above the FKGL target. KG-only was least readable on both metrics; RAG-only and GraphRAG were statistically comparable for FRE and FKGL, with GraphRAG producing modestly shorter responses (adjusted *p* = 0.043). The boxes indicate IQRs; the horizontal line indicates the median; the whiskers indicate 1.5 × IQR; the circles indicate outliers. FRE—Flesch Reading Ease (higher = more readable); FKGL—Flesch−Kincaid Grade Level (lower = more readable); IQR—interquartile range.

**Figure 9:**
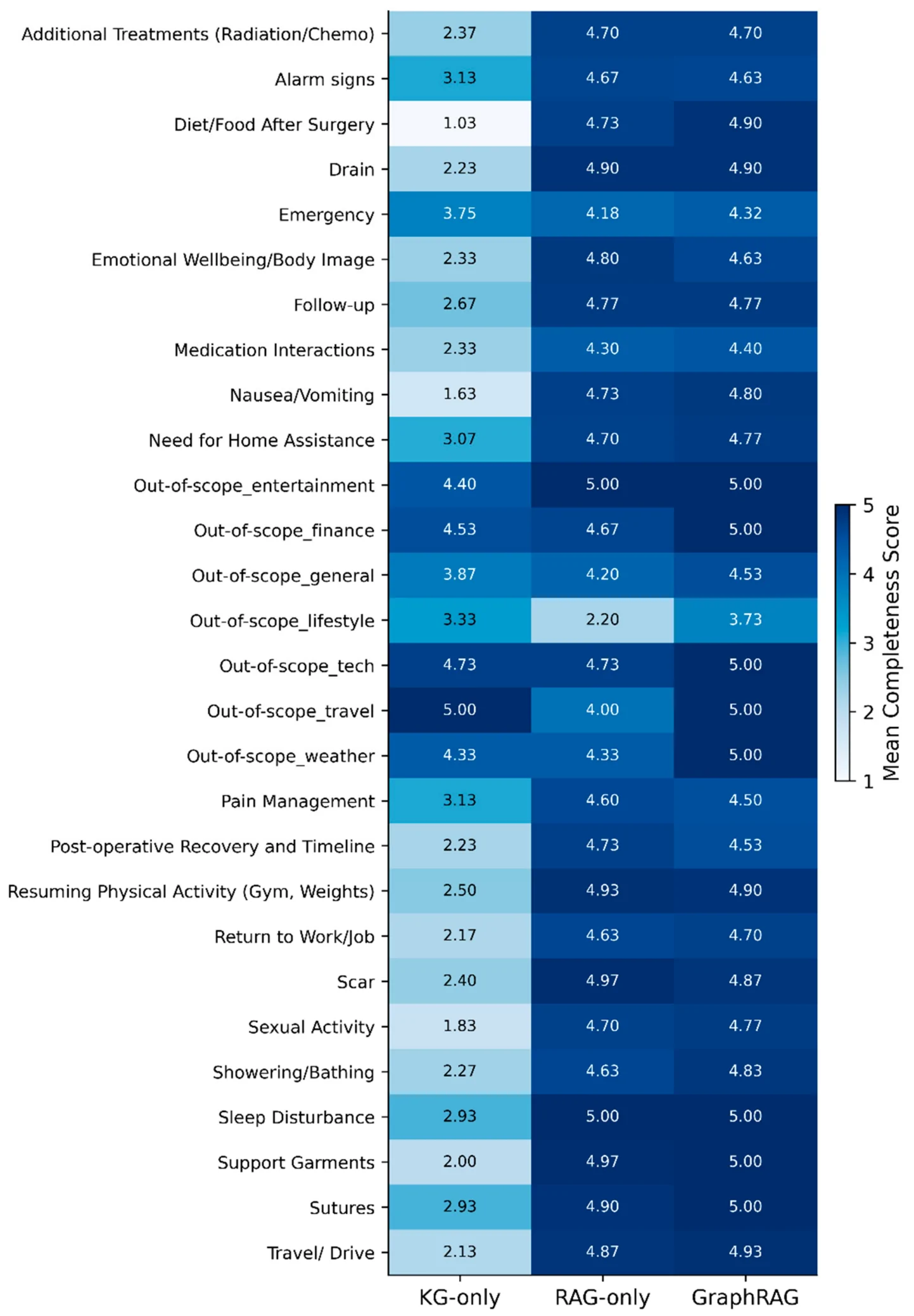
Category-wise clinician-rated completeness across KG-only, RAG-only, and GraphRAG. Mean completeness scores were calculated for each clinical and boundary-condition category using a 1–5 Likert scale rated by three blinded clinicians. KG-only showed greater category-level variability, whereas RAG-only and GraphRAG maintained consistently higher completeness across most domains.

**Table 1:** Composition of the 250-question postoperative evaluation benchmark by question group and category.

Question Group	Categories Included	*n*	%
Routine postoperative care	Pain management; wound/drain care; diet; medication interactions; activity; nausea/vomiting; showering; sleep; support garments; sexual activity; sutures; scar; return to work; travel/driving; emotional wellbeing; home assistance; follow-up; recovery timeline; alarm signs; additional treatments	200	80.0
Emergency-intent questions	Questions containing urgent or escalation-sensitive postoperative symptoms	20	8.0
Out-of-scope questions	Entertainment, finance, general knowledge, lifestyle, technology, travel, and weather queries outside postoperative care	30	12.0
Total		250	100.0

**Table 2:** Human-rated performance summary across the three retrieval architectures.

Metric	KG-Only	RAG-Only	GraphRAG	Overall_*p*	Effect_Size
Clinical Medical Accuracy	50.9% (95% CI: 44.3–57.4)	96.4% (95% CI: 93.0–98.1)	97.3% (95% CI: 94.2–98.7)	—	—
Safety/Refusal Accuracy	93.3% (95%CI: 78.7–98.2)	83.3% (95% CI: 66.4–92.7)	93.3% (95% CI: 78.7–98.2)	—	—
Overall Accuracy	56.0% (95% CI: 49.8–62.0)	94.8% (95% CI: 91.3–96.9)	96.8% (95% CI: 93.8–98.4)	<0.001	Cochran Q = 188.06
Completeness, mean (SD)	2.703 (1.442)	4.644 (0.784)	4.732 (0.661)	<0.001	W = 0.446
Completeness, median (range)	2.3 (1.0–5.0)	5.0 (1.0–5.0)	5.0 (1.0–5.0)	—	—
Relevance, mean (SD)	1.841 (0.852)	2.883 (0.367)	2.909 (0.328)	<0.001	W = 0.364
Relevance, median (range)	1.7 (0.3–3.0)	3.0 (1.0–3.0)	3.0 (1.0–3.0)	—	—

**Table 3:** Confusion matrix and derived composite classification metrics across the full benchmark (with 95% confidence intervals).

	KG-Only (N = 250)	RAG-Only (N = 250)	GraphRAG (N = 250)
**TP**	112	212	214
**TN**	28	25	28
**FP**	2	5	2
**FN**	108	8	6
**Overall accuracy**	56.0% (95%, 49.8–62.0)	94.8% (95%, 91.3–96.9)	96.8% (95%, 93.8–98.4)
**Clinical Medical Accuracy**	50.9% (95%, 44.3–57.4)	96.4% (95%, 93.0–98.1)	97.3% (95%, 94.2–98.7)
**Safety/Refusal Accuracy**	93.3% (95%, 78.7–98.2)	83.3% (95%, 66.4–92.7)	93.3% (95%, 78.7–98.2)
**Precision**	98.2% (93.8%, 99.5%)	97.7% (94.7%, 99.0%)	99.1% (96.7%, 99.7%)
**Recall**	50.9% (44.3%, 57.4%)	96.4% (93.0%, 98.1%)	97.3% (94.2%, 98.7%)
**F1**	67.1% (61.1%, 72.6%)	97.0% (95.3%, 98.6%)	98.2% (96.7%, 99.3%)

TP—true positive; TN—true negative; FP—false positive; FN—false negative. Classification precision—TP/(TP + FP); recall—TP/(TP + FN); F1 is the harmonic mean.

**Table 4:** RAGAS automated evaluation metrics across the three architectures.

Metric	KG-Only Mean (SD)	RAG-Only Mean (SD)	GraphRAG Mean (SD)	chi2 (Friedman)	*p*	Kendall’s W
**Context Precision**	0.6247 (0.4219)	0.7777 (0.3804)	0.7297 (0.3579)	102.681	**<0.001**	0.205
**Context Recall**	0.7031 (0.3225)	0.7958 (0.2835)	0.8507 (0.2332)	57.953	**<0.001**	0.116
**Answer Similarity**	0.8831 (0.0886)	0.9257 (0.0830)	0.9379 (0.0677)	139.197	**<0.001**	0.278

**Table 5:** Binary-normalized hallucination and safety panel across the three architectures.

Metric	KG-Only Mean (SD)	RAG-Only Mean (SD)	GraphRAG Mean (SD)	chi2 (Friedman)	*p*	Kendall’s W
**Factual Correctness**	0.4000 (0.4899)	0.8840 (0.3202)	0.8920 (0.3104)	203.932	**<0.001**	0.408
**Faithfulness**	0.2680 (0.4429)	0.7840 (0.4115)	0.8200 (0.3842)	209.158	**<0.001**	0.418
**Completeness**	0.2240 (0.4169)	0.7960 (0.4030)	0.8360 (0.3703)	242.862	**<0.001**	0.486
**Safety**	0.5360 (0.4987)	0.8560 (0.3511)	0.8520 (0.3551)	103.623	**<0.001**	0.207
**Hallucination-Free Score**	0.5640 (0.4959)	0.8680 (0.3385)	0.8920 (0.3104)	104.467	**<0.001**	0.209
**Hallucination Risk**	0.4360 (0.4959)	0.1320 (0.3385)	0.1080 (0.3104)	104.467	**<0.001**	0.209

**Table 6:** Retrieval diagnostics and response-behavior characteristics of KG-only, RAG-only, and GraphRAG.

Outcome	KG-Only	RAG-Only	GraphRAG
**Mean retrieval latency, s**	6.67	4.59	10.66
**Median retrieval latency, s**	6.47	4.57	11.00
**Mean retrieved chunk count**	7.81 (linked)	8.00	8.00 (base RAG)
**Mean retrieved graph-fact count**	13.63	—	13.63
**Mean selected graph-fact count**	—	—	11.75
**Mean KG-linked chunks**	—	—	7.81
**Mean graph-guided retrieved chunks**	—	—	10.00
**Mean final merged evidence count**	—	—	10.00
**Graph-guided retrieval rate**	—	—	100%
**Graph-suppression rate**	—	—	0.00
**RAG-anchor rate**	—	—	0.448
**Abstention rate**	0.008	0.000	0.000
**IDK rate**	0.004	0.000	0.000

## Data Availability

The full knowledge base cannot be publicly shared due to permission and licensing constraints associated with the source materials. The remaining data supporting the findings of this study are available within the article and its Supplementary Materials.

## Supplementary Materials

The following supporting information can be downloaded at: https://www.mdpi.com/article/10.3390/bioengineering13101097/s1, Table S1: Pairwise statistical comparisons between the three retrieval architectures (KG-only, RAG-only, GraphRAG) across all evaluated domains and variables. Raw *p*-values are reported alongside Bonferroni-adjusted *p*-values.
