# Supplementary material for: Graph-Enhanced Retrieval-Augmented Generation for Postoperative Patient Question Answering: A Comparative Evaluation of Knowledge Graph, Retrieval-Augmented Generation, and Hybrid GraphRAG Approaches
Source: Bioengineering (Basel). 2026 Sep 22;13(10):1097. doi: 10.3390/bioengineering13101097 (PMC13701952; doi:10.3390/bioengineering13101097)
Supplement: Supplementary file 1 [file bioengineering-13-01097-s001.zip › bioengineering-4564643-supplementary.pdf]

Supplementary Materials

Graph-Enhanced Retrieval-Augmented Generation for Postoperative Patient Question Answering: A Comparative Evaluation of Knowledge Graph, Retrieval-Augmented Generation, and Hybrid GraphRAG Approaches

Table S1. Pairwise statistical comparisons between the three retrieval architectures (KG-only, RAG-only, GraphRAG) across all evaluated domains and variables. Raw p-values are reported alongside Bonferroni-adjusted p-values.

Domain	Variable	Comparison	Test	n	Statistic	Effect_Type	Effect_Size	Raw_p	Adjusted p (Bonferroni)	Significance	Interpretation
Accuracy	Overall Accuracy (correct vs incorrect)	KG-only vs RAG-only	McNemar	250	OR = 0.044	OR (95% CI)	0.044 [0.017–0.114]	<0.001	<0.001	***	Favours RAG-only
Accuracy	Overall Accuracy (correct vs incorrect)	KG-only vs GraphRAG	McNemar	250	OR = 0.014	OR (95% CI)	0.014 [0.003–0.073]	<0.001	<0.001	***	Favours GraphRAG
Accuracy	Overall Accuracy (correct vs incorrect)	RAG-only vs GraphRAG	McNemar	250	OR = 0.091	OR (95% CI)	0.091 [0.005–1.644]	0.062	0.188	ns	No significant difference
Accuracy	Clinical Medical Accuracy (n = 220)	KG-only vs RAG-only	McNemar	220	OR = 0.005	OR (95% CI)	0.005 [0.000–0.080]	<0.001	<0.001	***	Favours RAG-only
Accuracy	Clinical Medical Accuracy (n = 220)	KG-only vs GraphRAG	McNemar	220	OR = 0.005	OR (95% CI)	0.005 [0.000–0.079]	<0.001	<0.001	***	Favours GraphRAG
Accuracy	Clinical Medical Accuracy (n = 220)	RAG-only vs GraphRAG	McNemar	220	OR = 0.200	OR (95% CI)	0.200 [0.010–4.166]	0.500	1.000	ns	No significant difference
Accuracy	Safety/Refusal Accuracy (n = 30)	KG-only vs RAG-only	McNemar	30	OR = 3.000	OR (95% CI)	3.000 [0.473–19.039]	0.375	1.000	ns	No significant difference
Accuracy	Safety/Refusal Accuracy (n = 30)	KG-only vs GraphRAG	McNemar	30	OR = 1.000	OR (95% CI)	1.000 [0.104–9.614]	1.000	1.000	ns	No significant difference
Accuracy	Safety/Refusal Accuracy (n = 30)	RAG-only vs GraphRAG	McNemar	30	OR = 0.143	OR (95% CI)	0.143 [0.007–2.766]	0.250	0.750	ns	No significant difference
Completeness	Completeness Score (1–5, mean of 3 raters)	KG-only vs RAG-only	Wilcoxon signed-rank	250	W = 571.0	r (effect size)	0.508	<0.001	<0.001	***	Large — Favours RAG-only
Completeness	Completeness Score (1–5, mean of 3 raters)	KG-only vs GraphRAG	Wilcoxon signed-rank	250	W = 49.5	r (effect size)	0.508	<0.001	<0.001	***	Large — Favours GraphRAG
Completeness	Completeness Score (1–5, mean of 3 raters)	RAG-only vs GraphRAG	Wilcoxon signed-rank	250	W = 791.0	r (effect size)	0.138	0.029	0.087	ns	No significant difference

Domain	Variable	Comparison	Test	n	Statistic	Effect_Type	Effect_Size	Raw_p	Adjusted p (Bonferroni)	Significance	Interpretation
Relevance	Relevance Score (0–3, mean of 3 raters)	KG-only vs RAG-only	Wilcoxon signed-rank	250	W = 250.5	r (effect size)	0.508	<0.001	<0.001	***	Large — Favours RAG-only
Relevance	Relevance Score (0–3, mean of 3 raters)	KG-only vs GraphRAG	Wilcoxon signed-rank	250	W = 91.5	r (effect size)	0.508	<0.001	<0.001	***	Large — Favours GraphRAG
Relevance	Relevance Score (0–3, mean of 3 raters)	RAG-only vs GraphRAG	Wilcoxon signed-rank	250	W = 57.0	r (effect size)	0.150	0.018	0.054	ns	No significant difference
RAGAS (automated)	Context Precision	KG-only vs RAG-only	Wilcoxon signed-rank	250	W = 1882.0	r (effect size)	0.453	<0.001	<0.001	***	Moderate — Favours RAG-only
RAGAS (automated)	Context Precision	KG-only vs GraphRAG	Wilcoxon signed-rank	250	W = 4414.5	r (effect size)	0.466	<0.001	<0.001	***	Moderate — Favours GraphRAG
RAGAS (automated)	Context Precision	RAG-only vs GraphRAG	Wilcoxon signed-rank	250	W = 7590.0	r (effect size)	0.190	0.003	0.008	**	Small — Favours RAG-only
RAGAS (automated)	Context Recall	KG-only vs RAG-only	Wilcoxon signed-rank	250	W = 2974.5	r (effect size)	0.264	<0.001	<0.001	***	Small — Favours RAG-only
RAGAS (automated)	Context Recall	KG-only vs GraphRAG	Wilcoxon signed-rank	250	W = 902.5	r (effect size)	0.469	<0.001	<0.001	***	Moderate — Favours GraphRAG
RAGAS (automated)	Context Recall	RAG-only vs GraphRAG	Wilcoxon signed-rank	250	W = 929.5	r (effect size)	0.278	<0.001	<0.001	***	Small — Favours GraphRAG
RAGAS (automated)	Answer Similarity	KG-only vs RAG-only	Wilcoxon signed-rank	250	W = 4089.0	r (effect size)	0.508	<0.001	<0.001	***	Large — Favours RAG-only
RAGAS (automated)	Answer Similarity	KG-only vs GraphRAG	Wilcoxon signed-rank	250	W = 2344.0	r (effect size)	0.508	<0.001	<0.001	***	Large — Favours GraphRAG
RAGAS (automated)	Answer Similarity	RAG-only vs GraphRAG	Wilcoxon signed-rank	250	W = 7492.0	r (effect size)	0.125	0.049	0.146	ns	No significant difference
Hallucination	Factual Correctness (norm)	KG-only vs RAG-only	Wilcoxon signed-rank	250	W = 630.0	r (effect size)	0.508	<0.001	<0.001	***	Large — Favours RAG-only
Hallucination	Factual Correctness (norm)	KG-only vs GraphRAG	Wilcoxon signed-rank	250	W = 335.0	r (effect size)	0.508	<0.001	<0.001	***	Large — Favours GraphRAG
Hallucination	Factual Correctness (norm)	RAG-only vs GraphRAG	Wilcoxon signed-rank	250	W = 94.5	r (effect size)	0.028	0.655	1.000	ns	No significant difference
Hallucination	Faithfulness (norm)	KG-only vs RAG-only	Wilcoxon signed-rank	250	W = 750.0	r (effect size)	0.508	<0.001	<0.001	***	Large — Favours RAG-only
Hallucination	Faithfulness (norm)	KG-only vs GraphRAG	Wilcoxon signed-rank	250	W = 535.5	r (effect size)	0.508	<0.001	<0.001	***	Large — Favours GraphRAG
Hallucination	Faithfulness (norm)	RAG-only vs GraphRAG	Wilcoxon signed-rank	250	W = 336.0	r (effect size)	0.089	0.160	0.480	ns	No significant difference

Domain	Variable	Comparison	Test	n	Statistic	Effect_Type	Effect_Size	Raw_p	Adjusted p (Bonferroni)	Significance	Interpretation
Hallucination	Completeness (norm)	KG-only vs RAG-only	Wilcoxon signed-rank	250	W = 820.0	r (effect size)	0.508	<0.001	<0.001	***	Large — Favours RAG-only
Hallucination	Completeness (norm)	KG-only vs GraphRAG	Wilcoxon signed-rank	250	W = 324.0	r (effect size)	0.508	<0.001	<0.001	***	Large — Favours GraphRAG
Hallucination	Completeness (norm)	RAG-only vs GraphRAG	Wilcoxon signed-rank	250	W = 273.0	r (effect size)	0.103	0.105	0.314	ns	No significant difference
Hallucination	Safety (norm)	KG-only vs RAG-only	Wilcoxon signed-rank	250	W = 695.5	r (effect size)	0.491	<0.001	<0.001	***	Moderate — Favours RAG-only
Hallucination	Safety (norm)	KG-only vs GraphRAG	Wilcoxon signed-rank	250	W = 689.0	r (effect size)	0.488	<0.001	<0.001	***	Moderate — Favours GraphRAG
Hallucination	Safety (norm)	RAG-only vs GraphRAG	Wilcoxon signed-rank	250	W = 272.0	r (effect size)	0.011	0.862	1.000	ns	No significant difference
Hallucination	Hallucination-Free Score	KG-only vs RAG-only	Wilcoxon signed-rank	250	W = 872.0	r (effect size)	0.463	<0.001	<0.001	***	Moderate — Favours RAG-only
Hallucination	Hallucination-Free Score	KG-only vs GraphRAG	Wilcoxon signed-rank	250	W = 577.5	r (effect size)	0.508	<0.001	<0.001	***	Large — Favours GraphRAG
Hallucination	Hallucination-Free Score	RAG-only vs GraphRAG	Wilcoxon signed-rank	250	W = 159.5	r (effect size)	0.072	0.257	0.771	ns	No significant difference
Hallucination	Hallucination Risk	KG-only vs RAG-only	Wilcoxon signed-rank	250	W = 872.0	r (effect size)	0.463	<0.001	<0.001	***	Moderate — Favours RAG-only
Hallucination	Hallucination Risk	KG-only vs GraphRAG	Wilcoxon signed-rank	250	W = 577.5	r (effect size)	0.508	<0.001	<0.001	***	Large — Favours GraphRAG
Hallucination	Hallucination Risk	RAG-only vs GraphRAG	Wilcoxon signed-rank	250	W = 159.5	r (effect size)	0.072	0.257	0.771	ns	No significant difference; direction favours GraphRAG
Readability	FRE Score (Flesch Reading Ease)	KG-only vs RAG-only	Wilcoxon signed-rank	250	W = 7503.0	r (effect size)	0.452	<0.001	<0.001	***	Favours RAG-only (higher FRE = more readable)
Readability	FRE Score (Flesch Reading Ease)	KG-only vs GraphRAG	Wilcoxon signed-rank	250	W = 6646.0	r (effect size)	0.500	<0.001	<0.001	***	Favours GraphRAG (higher FRE = more readable)
Readability	FRE Score (Flesch Reading Ease)	RAG-only vs GraphRAG	Wilcoxon signed-rank	250	W = 7852.0	r (effect size)	0.433	0.168	0.503	ns	No significant difference
Readability	FKGL Grade Level (Flesch-Kincaid)	KG-only vs RAG-only	Wilcoxon signed-rank	250	W = 8787.0	r (effect size)	0.381	<0.001	<0.001	***	Favours RAG-only (lower FKGL = more readable)
Readability	FKGL Grade Level (Flesch-Kincaid)	KG-only vs GraphRAG	Wilcoxon signed-rank	250	W = 8101.0	r (effect size)	0.419	<0.001	<0.001	***	Favours GraphRAG (lower FKGL = more readable)

Domain	Variable	Comparison	Test	n	Statistic	Effect_Type	Effect_Size	Raw_p	Adjusted p (Bonferroni)	Significance	Interpretation
Readability	FKGL Grade Level (Flesch-Kincaid)	RAG-only vs GraphRAG	Wilcoxon signed-rank	250	W = 8320.0	r (effect size)	0.407	0.451	1.000	ns	No significant difference
Readability	Word Count (words per response)	KG-only vs RAG-only	Wilcoxon signed-rank	250	W = 1589.5	r (effect size)	0.779	<0.001	<0.001	***	KG-only shorter responses
Readability	Word Count (words per response)	KG-only vs GraphRAG	Wilcoxon signed-rank	250	W = 1576.5	r (effect size)	0.780	<0.001	<0.001	***	KG-only shorter responses
Readability	Word Count (words per response)	RAG-only vs GraphRAG	Wilcoxon signed-rank	250	W = 6971.5	r (effect size)	0.482	0.014	0.043	*	GraphRAG shorter (adj. p = 0.043)

Note (revised): all nine accuracy comparisons in this table were recomputed from the per-question majority-consensus reviewer data (n = 250; clinical subset n = 220; out-of-scope subset n = 30). Odds ratios use the Haldane-Anscombe continuity correction where a discordant cell was zero, p-values are exact two-sided McNemar values, and adjusted p-values apply Bonferroni correction for three pairwise comparisons per outcome. The RAG-only versus GraphRAG overall-accuracy comparison is not statistically significant after correction (adjusted p = 0.188), superseding the previously reported value.
